# Influence of inflow directions and setting angle of inlet guide vane on hydraulic performance of an axial-flow pump

**DOI:** 10.1038/s41598-023-30511-4

**Published:** 2023-03-01

**Authors:** Duc-Anh Nguyen, Sang-Bum Ma, Sung Kim, Jin-Hyuk Kim

**Affiliations:** 1 Convergence Manufacturing System Engineering (Green Process and Energy System Engineering), University of Science & Technology, Daejeon, 34113 South Korea; 2 Carbon Neutral Technology R&D Department, Korea Institute of Industrial Technology, Cheonan, 31056 South Korea

**Keywords:** Engineering, Mechanical engineering

## Abstract

Inlet flow direction significantly affects the hydraulic performance of an axial-flow pump. Usually, the research papers ignore this phenomenon, resulting in discrepancies between simulation and experimental results. This study examines the influence of inflow direction in five cases (0%, 5%, 10%, 15%, and 30% pre-swirl intensities) to determine the relationship between the pre-swirl intensity and the hydraulic performance of the axial-flow pump. Based on this, changing the setting angle of the inlet guide vane (IGV) is proposed and thoroughly investigated to reduce the effect of inflow direction. In this study, the influence of clearances in IGV blades on hydraulic performance is also investigated in detail. Numerical simulations are performed using ANSYS–CFX and a shear stress transport reattachment modification (SST k-$\omega$) turbulence model with small y+ values at all walls. Specifically, the hydraulic performance curves and internal flow characteristics, including contours and streamlines, are assessed and analyzed. The inflow direction significantly impacts the hydraulic efficiency of the axial-flow pump. Increased pre-swirl intensity causes more loss in the IGV passage. The internal flow field and performance are not affected by the clearance at the hub and shroud of the IGV. However, the tip clearance of the impeller causes a decrease in hydraulic efficiency due to the tip leakage vortex. By adjusting the setting angle of the IGV, the efficiency and head gradually increase from a negative to a positive setting angle. Additionally, 30° is considered the critical setting angle for IGV.

## Introduction

Among the various types of pumps, the axial-flow pump can generate the largest flow rate with high efficiency and is widely applied in agriculture, irrigation, water supply, and drainage in factories, urban areas, etc. The internal flow field of an axial-flow pump is three-dimensional, complex, and unsteady because of the formation of many vortices, such as the tip leakage vortex (TLV)^1–5^, the horseshoe vortex^5^, secondary flow^5^, and trailing edge vortex^5^. In addition, there is a hydraulic loss due to cavitation^6–9^ and backward leakage flow through the impeller blade tip clearance^5^. They often cause vibration, noise, and impeller blade damage^10,11^.

Computational fluid dynamics (CFD) is an indispensable method for product research and development because of its usefulness, cost and time savings, and efficiency^12,13^. To record and analyze the phenomena occurring inside the pump, the most recent research utilizes CFD method and sometimes combines it with experimental tests. However, in most axial-flow pump studies using CFD method, the inflow direction before entering the inlet guide vane (IGV) is straight^14–17^. In fact, this inflow direction is not straight; instead, it swirls around the rotational shaft of the pump because of the effect of the impeller^18^. Therefore, a difference between simulation results and experimental results is inevitable while doing the practical test^19–21^. Currently, there are no specific research articles stating the effect of inflow direction to verify this occurrence. Consequently, in this study, the change in inflow direction is thoroughly investigated to confirm their influence on the hydraulic performance of axial-flow pumps.

At design conditions, the axial-flow pump can stably operate for a long time with high performance and no phenomena. Notwithstanding, at low flow rate conditions, internal flow physics are unstable, and the stall phenomenon occurs, causing a sharp drop in hydraulic performance^3,15,16,22^. When the pump operates in the saddle zone, loud noises and severe vibrations are produced. In addition, it reduces the head and significantly impacts the safe and stable operation of the pump, as well as its long-term durability. To improve this phenomenon and efficiency at the off-design point, the setting angle of the impeller is adjusted by a rotating mechanism^23,24^. This strategy has contributed to a significant improvement in the hydraulic performance of the pump. However, to do this, the impeller structure of the pump must be highly complex and comprised of many components. In addition, because the impeller is a moving part, the impeller with a rotating mechanism is less sturdy than the integrally cast impeller^24^. In this study, changing the setting angle of the IGV to restrict the influence of inflow direction is discussed to increase system flexibility and rigidity. This method is novel today.

To do this, it is necessary to create clearance in the IGV. There have been many research papers on clearance, and the results show that clearance significantly affects the internal flow characteristics of the pump, especially in the impeller. Feng et al.^14^ performed a numerical simulation to determine the impact of different tip clearances on pressure fluctuations. The results showed that as tip clearance increased, pressure fluctuation within the impeller increased. Han et al.^25^ examined the impact of tip clearance on the hydraulic performance of the axial-flow water-jet propulsion pump in a separate study. In that study, the saddle zone decreased when the tip clearance increased, and the high efficiency area significantly narrowed. In an independent study, Lin et al.^26^ concluded that when the tip clearance increases, the hydraulic performance decreases under each flow rate condition, and the vortex widely spreads inside the impeller part. Shen et al.^4^ demonstrated that the width of the tip clearance significantly affects the structure and development of tip leakage flow. Most studies on tip clearance in the impeller conclude that as tip clearance increases, hydraulic performance decreases. However, no studies show the influence of IGV clearance. Consequently, in this work, the clearances in the IGV are investigated in detail to determine their effect.

The appearance of the IGV reduces turbulence in front of the impeller and produces a smooth flow when operating at the off-design point. Yang et al.^27^ investigated numerical simulation for adjustable IGV. The results showed that the setting angle of an IGV significantly affects its hydraulic performance. Zhang et al.^28^ studied the influence of the inflow condition on the efficiency of the axial-flow pump. The head generally increases when vortex generators (VG) are present. According to the article, the way to set the VG’s setting angle is changed compared to the normal way (straight VG); however, the different angles are not investigated in detail.

In previous research, in-depth studies were not conducted on the correlation between the inlet flow direction and the setting angle of the IGV. In this study, the inflow directions in front of the IGV are therefore investigated using numerical analysis. To reduce the influence of the inflow direction, changing the setting angle of the IGV is proposed and thoroughly analyzed. To change the setting angle of the IGV, clearances must be created at the hub and shroud. In this work, the influence of the IGV clearances is also investigated in detail, along with the tip clearance of the impeller.

## Hypotheses and theoretical analysis

### Pre-swirl flow

**Figure 1:**
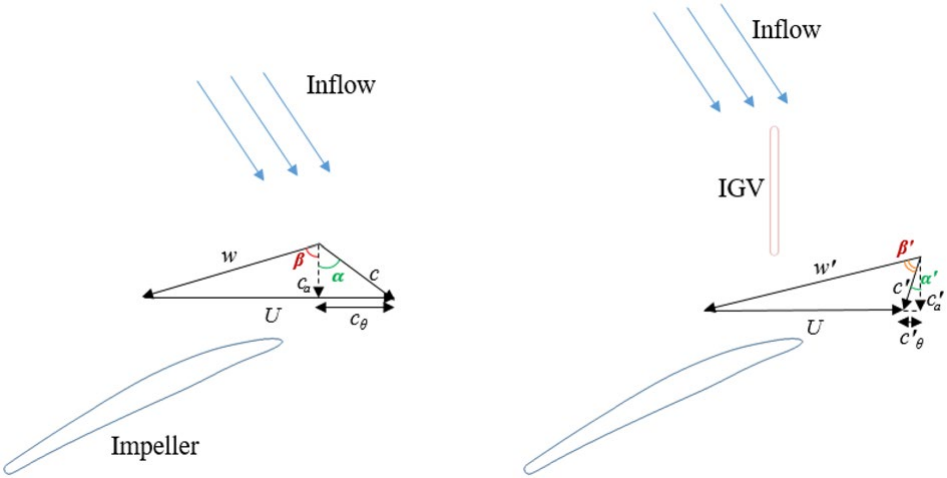
Inflow direction and velocity triangle.

In most axial-flow pump studies using the CFD method, the inlet flow direction before entering the IGV is straight compared to the rotating shaft^14–16^. In reality, under the effect of the impeller, this flow direction swirls around the rotating shaft of the axial pump^18^. Therefore, it causes a difference between the simulation and the experimental results. In other words, the hydraulic performance and internal flow field are directly affected by pre-swirl flow in front of the impeller due to the change in the velocity components. The velocity triangle at the leading edge (LE) of the impeller is shown in Fig. 1. In the absence of IGV, a high absolute flow angle results in a considerable mismatch between the flow angle and blade angle at the LE of the impeller, leading to a drop in efficiency. The IGV can reduce the influence of pre-swirl flow by changing the flow direction before entering the impeller. With the presence of IGV, the absolute flow angle decreases compared to the case without IGV. This smoothens the interaction between the inflow and the impeller, resulting in less loss and improved performance.

The flow angle in front of the IGV is defined as follows:

1 $${\text{tan}}\delta = \frac{{V}_{c}}{{V}_{a}},$$

where $\delta$, ${\mathrm{V}}_{\mathrm{c}}$, and ${V}_{a}$ denote the angle between inflow direction and the rotating shaft, swirl’s circumferential velocity component, and swirl’s axial velocity component, respectively.

Before entering the IGV, fluid consists of only two velocity components: circumferential and axial. At each flow rate condition, the change in velocity results in a change in pre-swirl intensity, which affects the flow direction. With the increase in flow rate, the flow angle ($\delta$) gradually approaches zero, and the flow direction becomes straighter relative to the rotating shaft because of the decrease in the swirl’s circumferential velocity component (${V}_{c}$) and the increase in the swirl’s axial velocity component (${V}_{a}$). The pre-swirl intensity is defined according to the percentage value of the circumferential velocity component. With the increment in pre-swirl intensity, the increase in the swirl’s circumferential velocity component (${\mathrm{V}}_{\mathrm{c}}$) results in the enhancement of flow velocity, and fluid swirls around the rotating shaft, causing many losses due to viscosity and incompatibility between flow angle and blade angle at IGV’s LE. Therefore, the hydraulic performance significantly reduces when increasing the pre-swirl intensity.

### Clearances in the blade

The influence of the clearances in the IGV is supposed to be negligible as the IGV is a stationary part, and the velocity in the IGV part is relatively not complicated. Therefore, the hydraulic loss caused by clearance in the IGV is negligible. However, as many research articles have demonstrated, the loss caused by the tip clearance of the impeller is significant. It directly affects the internal flow characteristics and the efficiency of the axial-flow pump. The hydraulic loss is primarily due to the TLV at the tip of the impeller. The formation of this vortex comes from the difference in pressure between the two sides of the blade, resulting in the fluid overflowing from the pressure surface (PS) to the suction surface (SS) and then forming the vortex. Therefore, the clearance should be considered small enough to limit fluid leakage through the clearance.

### Setting angle of the IGV

The pre-swirl flow in front of the impeller can be handled efficiently by changing the IGV’s setting angle. To improve efficiency under off-design conditions, the basic idea is to change the flow angle at the LE of the impeller to create the best conditions for the flow approaching the impeller. The highest performance is achieved when the flow angle and blade angle of the impeller are compatible. Consequently, the best method is to change the IGV’s setting angle.

**Figure 2:**
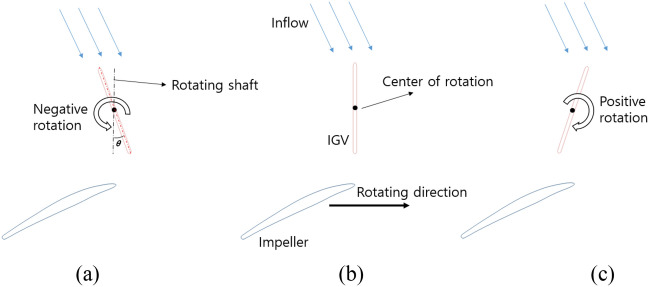
Schematic of the IGV’s setting angle. (**a**) Negative angle, (**b**) reference, and (**c**) positive angle.

The schematic of the IGV’s setting angle is shown in Fig. 2. In the reference model, the IGV’s setting angle equals zero. When rotating the IGV clockwise, the IGV’s position is denominated as a positive angle; otherwise, it is denominated as a negative angle. In the case of a positive setting angle, there is a certain loss in the IGV part due to the mismatch between the flow angle and the blade angle of the IGV. However, the fluid, after flowing through IGV, is redirected, creating a good condition for the interaction between the fluid and the impeller. As a result, hydraulic performance increases. On the contrary, with the negative setting angle, the flow approaches the IGV favorably. However, the high absolute flow angle at the LE of the impeller results in a decrease in performance, thereby reducing the function of IGV.

## Methodology

### Experimental setup

**Figure 3:**
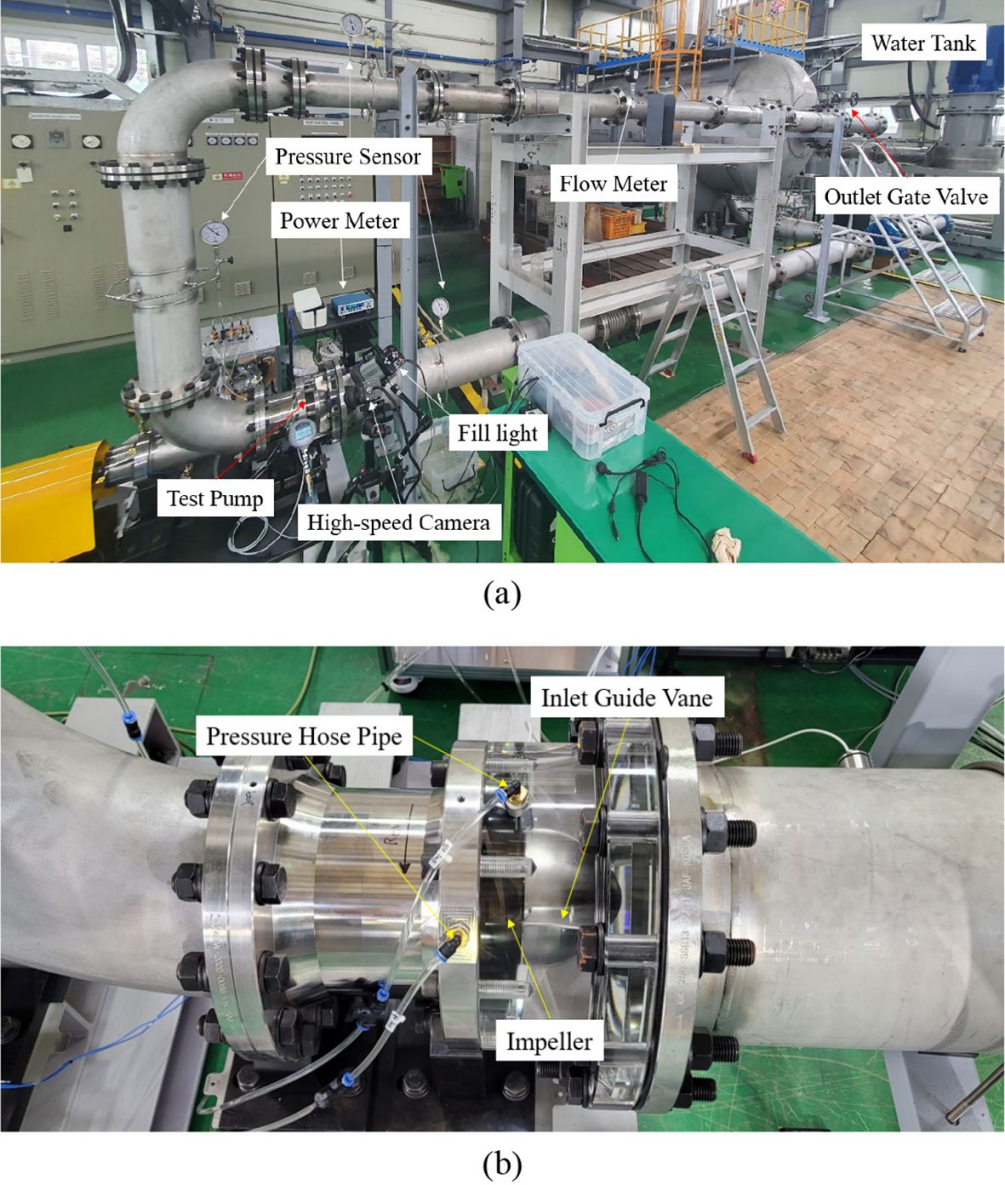
Experimental test of the axial-flow pump. (**a**) Test system, (**b**) Test pump.

The geometry of the axial-flow pump used in this study was tested at the Korea Institute of Machinery and Materials, Korea. The test rig’s system for the axial-flow pump is shown in Fig. 3. The experimental test was performed in a closed-loop test system with an impeller, and IGV chambers were made transparent to facilitate flow observation in the future with optimal models. The test system includes an electric motor, power meter, inlet pipeline system, outlet pipeline system, flow meter, outlet gate valve, water tank, pressure sensor, high-speed camera, fill light, and test pump. There are three pressure measuring devices, one mounted at the inlet and two at the outlet. Each pressure measuring device consists of four pressure hose pipes around the pipeline. In addition, two pressure hose pipes are located on the pipeline at the LE and trailing edge of the impeller to measure pressure fluctuations. The flow meter device is set far from the pump to receive a stable signal. To maintain the stable flow, the inlet and outlet of the axial-flow pump were extended away from the impeller to avoid interference with the backflow. A measuring system set behind the pipeline system is connected to the motor shaft to measure the shaft power. The phenomena occurring inside the pump are collected through a high-speed imaging system, including a fill light and a high-speed camera. The hydraulic performance data of the axial-flow pump at different flow rates was collected and processed through the data acquisition system. The operation ranges and uncertainty values of the main instruments are listed in Table 1.

**Table 1:** Accuracy of measuring instruments.

Instrument	Range	Uncertainty (%)
Absolute pressure transducer	0–200 kPa	$\pm$ 0.25
Differential pressure transducer	0–500 kPa	$\pm$ 0.2
Rotational sensor	0–20,000 r/min	$\pm$ 0.02
Flowmeter	0–900 m^3^/h	$\pm$ 0.2
Torque meter	0–200 Nm	$\pm$ 0.2

### Numerical analysis method

CFD^12^ is an effective method in product research and development because of its simplicity, cost and time savings, and accuracy in prediction. Much of the complicated internal flow physics occurring inside the pump cannot be observed only with experimental tests. However, the upgrading and improvement in the predictive ability of the CFD method make it easier. In the present study, to analyze the internal flow field and hydraulic characteristics of the axial-flow pump, the ANSYS–CFX–19.0 commercial code software is employed. Numerical simulations are obtained by using the three-dimensional (3D) steady and unsteady Reynolds-averaged Navier–Stokes equations (RANS and URANS) for 3D incompressible and isothermal flow. These RANS equations are discretized by using the finite volume method. The governing equations, including the continuity equation (Eq. 2) and the momentum equation (Eq. 3), can be written as follows:

2 $$\frac{\partial \rho }{\partial t}+ \frac{\partial \left(\rho {u}_{i}\right)}{\partial {x}_{i}}=0,$$

3 $$\rho \left[\frac{\partial ({u}_{i})}{\partial t}+ \frac{\partial ({u}_{i}{u}_{j})}{\partial {x}_{j}}\right]= -\frac{\partial p}{\partial {x}_{i}}+ \frac{\partial }{\partial {x}_{j}}\left[\mu \frac{\partial {u}_{i}}{\partial {x}_{j}}- \rho \overline{{u }_{i}^{^{\prime}}{u}_{j}^{^{\prime}}} \right],$$

where − $\rho \overline{{u }_{i}^{^{\prime}}{u}_{j}^{^{\prime}}}$, $\rho$, ${u}_{i}$, $\mu$, and $p$ are the Reynolds stress, the density, the velocity component in direction $i$, the turbulent viscosity, and the pressure, respectively.

### Turbulence model

To observe in detail the phenomena occurring near the wall regions, the shear stress transport reattachment modification (SST $k-\omega$) turbulent model is employed in this study. The SST turbulent model developed by Menter^29^ can relatively accurately predict the flow separation point occurring inside the pump with adverse pressure gradients. However, it is a well-known deficiency in all turbulent models that the reattachment position is often predicted too far downstream compared to what is observed in experiments. The reason is the under-prediction of turbulent stresses in the separated shear layers, and no turbulent model can predict this flow without modification. To improve this, a modification to the SST turbulent model has been developed to improve the predictive ability of the reattaching boundary layers by adding the source term “${P}_{reattach}$” in the transport equation (Eq. 4) for the turbulence kinetic energy^30^. It has no adverse effects on the accuracy of the SST model for attached and weakly separated boundary layers. Moreover, the SST model with the reattachment modification can result in a significant improvement in the predicted stall mass flow. The shear stress transport reattachment modification (SST $k-\omega$) turbulent model can be written as follows:

4 $$\frac{\partial (\rho {k}_{i})}{\partial t}+ \frac{\partial (\rho \overline{{U }_{j}}k)}{\partial {x}_{j}}= {P}_{k}- {\beta }^{*}\rho k\omega + \frac{\partial }{\partial {x}_{j}}\left[\left(\mu + \frac{{\mu }_{t}}{{\sigma }_{k}}\right)\frac{\partial k}{\partial {x}_{j}}\right]+ {P}_{reattach},$$

5 $$\frac{\partial (\rho \omega )}{\partial t}+ \frac{\partial (\rho {u}_{i}\omega )}{\partial {x}_{j}}= \frac{\gamma }{{\vartheta }_{t}}{P}_{k}- \beta \rho {\omega }^{2}+ \frac{\partial }{\partial {x}_{j}}\left[\left(\mu + {\sigma }_{\omega }{\mu }_{t}\right)\frac{\partial \omega }{\partial {x}_{j}}\right]+2\left(1- {F}_{1}\right)\frac{\rho {\sigma }_{{\omega }^{2}}}{\omega }\frac{\partial k}{\partial {x}_{j}}\frac{\partial \omega }{\partial {x}_{j}},$$

6 $${\mu }_{t}=\mathrm{min}\left(\frac{\rho k}{\omega }, \frac{\rho {a}_{1}{k}_{u}}{S{F}_{2}}\right),$$

7 $${P}_{reattach}= {P}_{k}.min\left[4max\left(0,\frac{min\left({S}^{2}, {\Omega }^{2}\right)}{0.09{\omega }^{2}}-1.6\right), 1.5\right],$$

8 $$\varnothing = {F}_{1}{\varnothing }_{1}+\left(1- {F}_{1}\right){\varnothing }_{2},$$

where $k$, ${P}_{k}$, $\omega$, $\mu$, ${\mu }_{t}$, ${\vartheta }_{t}$, S, $\Omega$, and $\varnothing$ are the turbulence kinetic energy, the production term, the specific turbulence dissipation, the molecular dynamic viscosity, the turbulent eddy viscosity, the turbulent kinematic viscosity, the invariant measure of the strain rate, the vorticity magnitude, and the constant, respectively.

The SST turbulence model takes the strength of k − $\omega$ and k − $\varepsilon$ models with a blending function (F1) which creates a smooth transition between these two models. The k − $\omega$ turbulence model analyzes the phenomena occurring at near-wall surfaces, and the k -$\varepsilon$ turbulence model calculates the bulk flow region for capturing the turbulent flow. The wall function is automatically applied in the calculating procedure for a smooth transition between these two turbulent models. The constants $\beta$, ${\sigma }_{k}$, ${\sigma }_{\omega }$ are calculated by a blend from the corresponding constants via Eq. (8). The constants are ${\beta }_{1}=0.075$, ${\beta }_{2}=0.0828$, ${\sigma }_{k1}=0.85$, ${\sigma }_{k2}=1.0$, ${\sigma }_{\omega 1}=0.5$, ${\sigma }_{\omega 2}=0.856$, ${\beta }^{*}=0.09$^29^.

### Cavitation model

Cavitation is basically a phase transition from liquid to vapor. Assuming that the cavitation flow behaves as a single flow with two phases having the same velocity and pressure fields, a homogeneous approximation to the two-phase flow (liquid/vapor) is employed. The Rayleigh–Plesset cavitation model is used to solve the URANS equations to eddy viscosity models that are used to describe the multi-phase flow. It served as the foundation for the rate equation governing the production of vapor and condensation^31^. The mass transport equation for the cavitation model can be described as follows:

9 $$\frac{\partial {\rho }_{v}{\alpha }_{v}}{\partial t}+ \nabla .\left({\rho }_{v}{\alpha }_{v}\overline{{u }_{i}}\right)= {A}_{e}+ {S}_{e},$$

where ${\rho }_{v}$ and ${\alpha }_{v}$ stand for the vapor phase density and the vapor volume fraction. ${A}_{e}$ and ${S}_{e}$ are source terms derived from the cavitation model. These source terms take into consideration cavitation’s mass transfer between the liquid and vapor phases. It can be described as follows:

10 $${A}_{e}= {F}_{v}\frac{3{r}_{n}(1-{\alpha }_{v}){\rho }_{v}}{{R}_{B}}\sqrt{\frac{2}{3}\frac{Max({P}_{v}-P,0)}{{\rho }_{l}}},$$

11 $${S}_{e}= {F}_{c}\frac{3{\alpha }_{v}{\rho }_{v}}{{R}_{B}}\sqrt{\frac{2}{3}\frac{Max({P}_{v}-P,0)}{{\rho }_{l}}},$$

where ${R}_{B}$, ${r}_{n}$, ${F}_{v}$, ${F}_{c}$, ${P}_{v}$, and ${\rho }_{l}$ denote bubble radius, the nucleation site volume fraction, the evaporation coefficient, the condensation coefficient, the vapor pressure, and the liquid density.

12 $${P}_{v}= {P}_{s}+ \frac{{P}_{t}}{2},$$

13 $${P}_{t}=0.39\left(1-{\alpha }_{v}\right){\rho }_{l}k,$$

where ${P}_{s}$ and ${P}_{t}$ represent the saturation vapor pressure and turbulent pressure fluctuation.

The internal flow field in the axial pump is three-dimensional, complex, and strongly unsteady flow. It is possible for the turbulent flow to cause a localized drop in pressure below the vapor pressure, which will quickly result in cavitation. Consequently, the effect of the cavitation phenomenon is considered in this study.

## Design and computational model

### Axial-flow pump model

**Figure 4:**
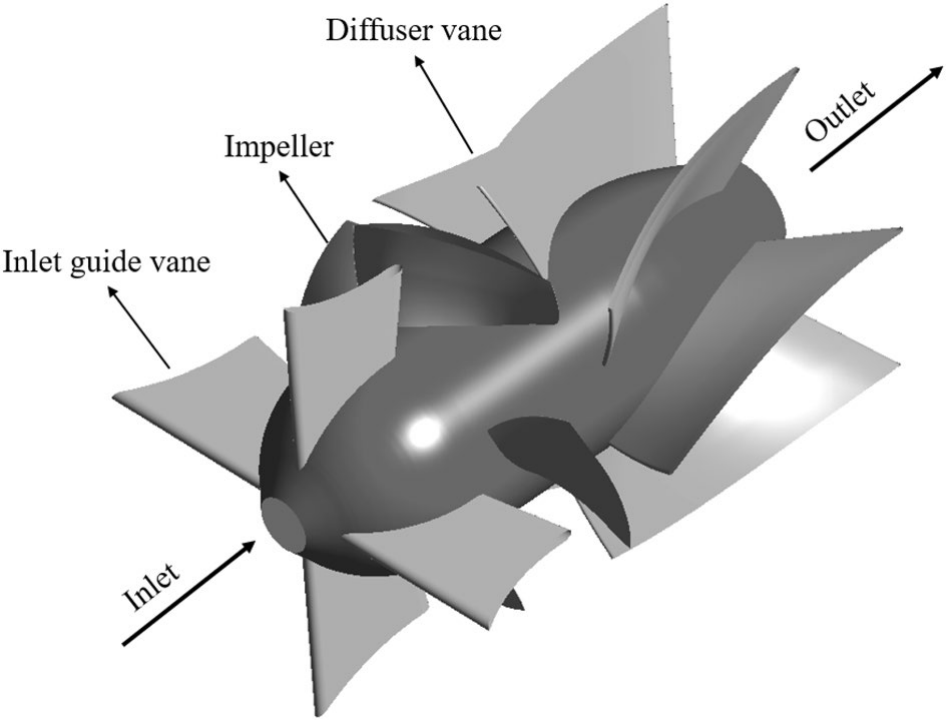
3D geometry of the axial-flow pump.

In this study, 3D numerical simulations are performed on the axial-flow pump with a specific speed of 1204 which is calculated by Eq. (14). The 3D geometry of the axial-flow pump evaluated in this study is shown in Fig. 4. The hydraulic components in the model consist of four stationary IGV blades, four rotating impeller blades, and seven stationary diffuser vane blades (DV). The tip clearance considered in the impeller blade is 0.0054 times the diameter of the impeller. The ratio of the impeller hub to shroud, which is defined as the impeller diameter ratio, is 0.2703. The model was scaled down eight times to the actual model. To prevent interference with the flow field inside the pump and observe fully developed turbulent flow, the inlet and outlet sections are extended to approximately four and five times the diameter of the impeller blade, respectively. The design flow rate coefficient (Eq. 15), head coefficient (Eq. 16), and rotational speed coefficient (Eq. 17) are 0.4319, 1.5841, and 0.7893, respectively, as shown in Table 2. Ideally, all passages should be modeled in the computational domain to examine the true internal flow field and to detect any asymmetry of the flow. However, most simulations of the flow field in the axial pump take advantage of the geometric symmetry, in this work, the computational domain is only constructed with one IGV passage, one impeller passage, and two DV passages with a pitch ratio of 1.00: 1.00: 1.14. To optimize the calculating accuracy and to cut down the profile scaling, this pitch ratio should be close to 1.0^32^^.^ As a result, two DV passages are modeled in the computational domain.

14 $${n}_{q}=n\left[rpm\right]\frac{{Q}^{0.5} [\frac{{m}^{3}}{min}]}{{H}^{0.75} [m]},$$

15 $$\varphi = \frac{Q}{n{D}^{3}},$$

16 $$\psi = \frac{gH}{{n}^{2}{D}^{2}},$$

17 $$\phi = \frac{nD}{\sqrt{gH}},$$

where $n$, *Q*, $\mathrm{H}$, *D*, and $g$ are the rotational speed, flow rate, head, impeller blade diameter, and gravitational acceleration, respectively.

**Table 2:** Specifications of axial-flow pump model.

Parameter	Value
Specific speed	1204
Impeller diameter ratio	0.2703
Design rotational speed coefficient	0.7893
Design head coefficient	1.5841
Design flowrate coefficient	0.4319
Number of inlet guide vanes (EA)	4
Number of impeller blades (EA)	4
Number of diffuser vanes (EA)	7

Previously, Fig. 1 illustrates the inflow direction with the presence of pre-swirl flow. In the case of investigated pre-swirl intensity, the IGV is fixed in the reference position with $\theta =0^\circ$. The pre-swirl intensities investigated in this work include 0%, 5%, 10%, 15%, and 30%. The flow direction in front of the IGV changes due to the effect of the pre-swirl intensity. With the increase of pre-swirl intensity, the inflow becomes more inclined with respect to the rotating shaft because of the enhancement in the swirl component of circumferential velocity and the decrease in the swirl component of axial velocity.

To adjust the IGV’s setting angle, creating the clearance at the hub and the shroud of the IGV is crucial. In addition, to facilitate the adjustment of the IGV, the biggest clearances should be determined at the hub and the shroud. In this study, the biggest setting angle is 30°. For this reason, to rotate an angle of 30° around the axis of IGV, the biggest clearance at the hub and the shroud are 0.0124 and 0.0076 times the impeller diameter, respectively. Based on these clearances, the three investigated cases include: without all gaps (reference case), only impeller gap, and all gaps (meaning: impeller gap, and IGV’s hub and shroud gaps).

**Figure 5:**
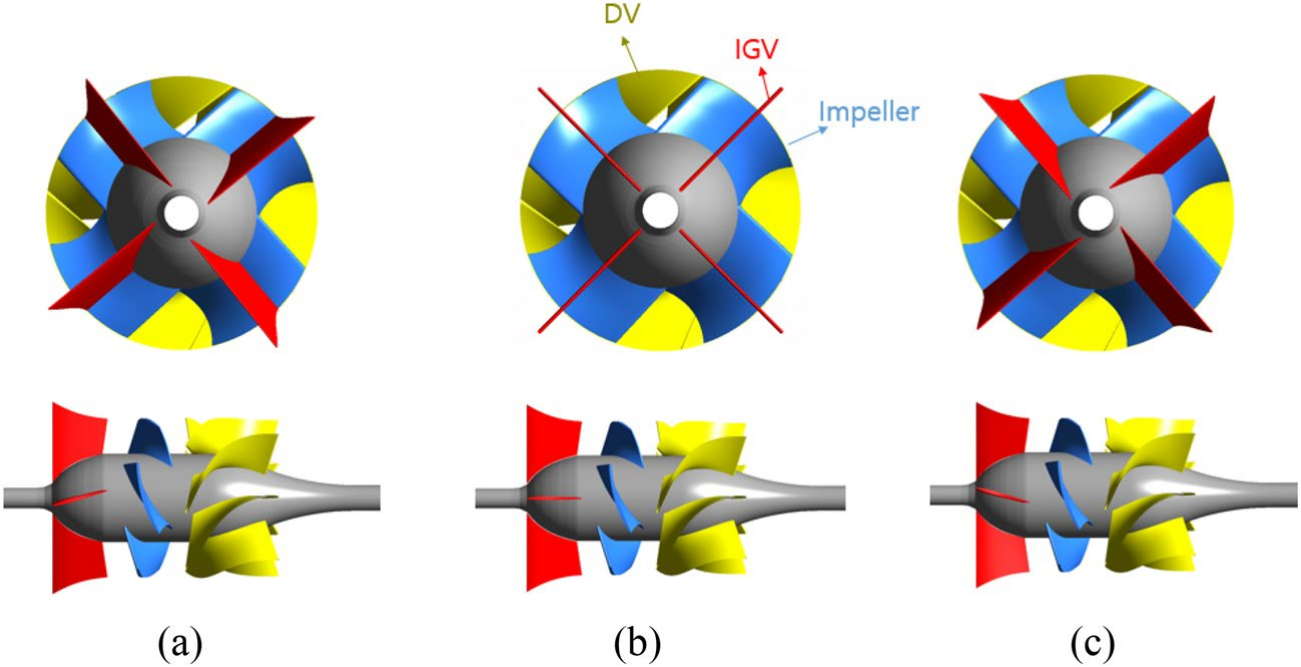
Front view and side view of axial-flow pump. (**a**) Negative IGV’s setting angle, (**b**) reference model, and (**c**) positive IGV’s setting angle.

Figure 2 illustrates a two-dimensional (2D) design for changing IGV’s setting angle. The center of rotation is the middle point of the camber line at the hub and the shroud of the IGV airfoil profile. The IGV’s setting angle $(\theta )$ is defined by the angle between the IGV’s camber line and the rotating shaft of the axial-flow pump. The setting angle $(\theta =0^\circ )$ was assumed as the reference position of IGV. In the present study, the internal flow field of the axial-flow pump with five different IGV’s setting angles is analyzed in detail and the range of the setting angle is from − 30$^\circ$ to 30$^\circ$. In this study, the flow visualization and behavior of the inlet flow direction are closely analyzed through experimental and numerical results. After obtaining the suitable inflow direction, the numerical scheme for the clearance and IGV’s setting angle analysis is also established. The 3D geometries of the axial-flow pump with different setting angles are depicted in Fig. 5.

### Boundary conditions

The boundary conditions are established using ANSYS–CFX Pre with working fluid was single-phase water at 25$^\circ{\rm C}$. The adiabatic and no-slip conditions are applied to all the wall regions. The periodic conditions are employed at the sides of each passage. The stage condition (mixing plane) is used to connect two different interfaces between the stationary and rotating domains. The total pressure and the mass flow rate are set at the inlet and outlet of the axial-flow pump, respectively. In the rotating part, the alternative rotation model is used to reduce the production of false swirls that occur when the grid is very coarse and/or the fluid motion is not well aligned with the rotating frame. The General Grid Interface is utilized to transfer data at the interface of domains using an intersection algorithm^32^. High-resolution is employed to discretize the convection terms^32^ and the turbulence numeric is the first order. A physical time scale is employed according to Eq. (18) to speed up the convergence process^31^. To confirm the convergence, the efficiency, total head, and flow rate functions are monitored in the CFX-Solver Manager with the fluctuation of less than 0.5% during 100-time steps. Moreover, the Root Mean Square value of the residuals, which is established for the time-dependent convergence criteria, is set at 10^−6^.

18 $$t=\frac{1}{10\omega n} \left(s\right),$$

where $t$, $\omega$, and $n$ denote as the physical time scale, the angular velocity of the blade, and the number of impeller blade.

To accelerate the convergence time in unsteady cavitation flow simulation, the unsteady numerical simulation is initialized with the steady numerical result. The saturation pressure of the water vapor and the mean bubble diameter at 25 $^\circ{\rm C}$ are set as 3170 (Pa) and $2 \times {10}^{-6}$ (m), respectively. For unsteady cavitation simulation, water and water vapor volume fractions are taken to be 1 and 0, respectively. In the unsteady simulation, a transient rotor/stator is employed for the interfaces between the stationary and rotating impeller. The full simulation time (T) is roughly 0.234 s, which is set to 10 times the rotation period of the impeller ($\mathcal{T}$). The time step is established at $2\times {10}^{-4}$ s, which is the time required for the impeller to rotate 3°. Four coefficient loops are established for each time step for further convergence.

### Mesh generation and dependence test

**Figure 6:**
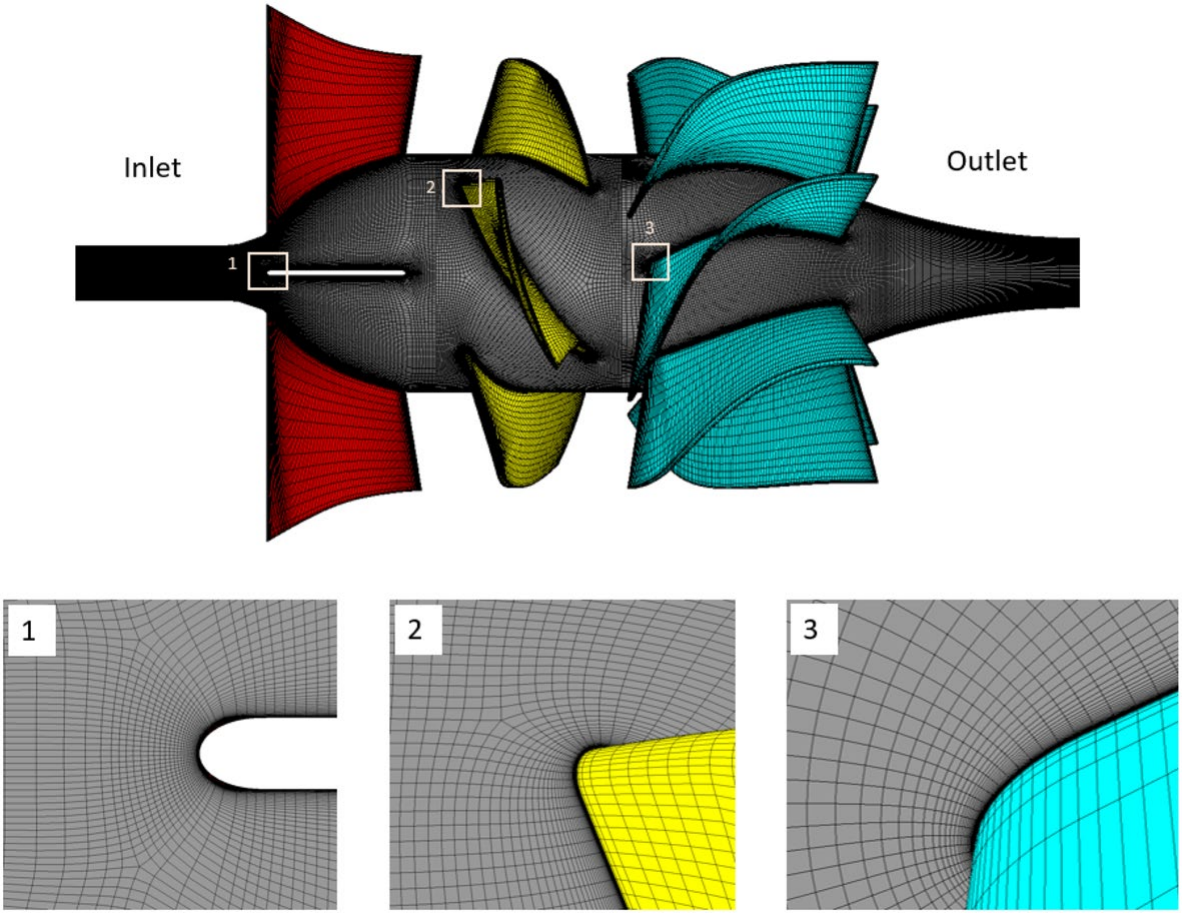
Grid system of the axial-flow pump.

Grid systems with a hexahedral structure are established based on the Turbogrid module of the ANSYS 19.0 software. Figure 6 depicts the numerical grid system of the axial-flow pump model. The grid is refined by multilayers of prism mesh applied to wall areas such as blade surfaces, hubs, and shrouds to guarantee that the y^+^ averaged value on these walls is less than two. The grid convergence is sequentially verified at the impeller, IGV, and DV passages. Therefore, after obtaining the optimal grid for impeller passage, this grid system is employed to find the optimal grid for IGV passage. The optimal grid system for the DV passage is determined using the ideal grid of the impeller and IGV passages.

The quality of the grid system significantly affects the convergence, the discretization error, and the accuracy of the result. Besides, the number of nodes also depends on the calculating ability of the computer. For these reasons, investigating grid independence is crucial to finding the most optimal grid. The grid convergence index method (GCI), developed by Celik et al.^33^, is employed to guarantee the independence of the grid. Three different grids (the course, medium, and fine grids) are used in the GCI method to estimate the numerical uncertainty because of discretization error. The extrapolations are carried out with a grid refinement factor higher than 1.3. The grid independence test is performed at the best efficiency point (BEP). The efficiency is selected as the key variable (η). The efficiency values in Table 3 indicate monotonic convergence according to the increase in the number of nodes. According to Celik et al.^33^, the index values of the essential variables are recommended to be less than 1%. Consequently, the low values of the GCI (${GCI}_{fine}^{21}$) and extrapolated relative error (${e}_{ext}^{21}$) indicate that the created grid systems are optimal and further grid refinement is not necessary.

**Table 3:** Grid independence test.

	Impeller only (No. of node of impeller passage)	IGV and impeller (No. of node of IGV passage)	Full component (No. of node of DV passage)
N_1_	666,773	550,272	571,540
N_2_	265,681	248,100	256,250
N_3_	115,260	409,164	114,500
r_21_	1.359	1.304	1.307
r_32_	1.321	1.315	1.308
${\upeta }_{1}/{\upeta }_{1}$	1.00	0.976	1.043
${\upeta }_{2}/{\upeta }_{1}$	0.993	0.975	1.042
${\upeta }_{3}/{\upeta }_{1}$	0.972	0.957	1.026
p	3.769	9.199	9.123
${e}_{ext}^{21}$	0.00325	0.00014	0.00012
${\mathrm{GCI}}_{medium}^{32}$	0.01412	0.00197	0.00178
${\mathrm{GCI}}_{\mathrm{fine}}^{21}$	0.00407	0.00017	0.00016

**Figure 7:**
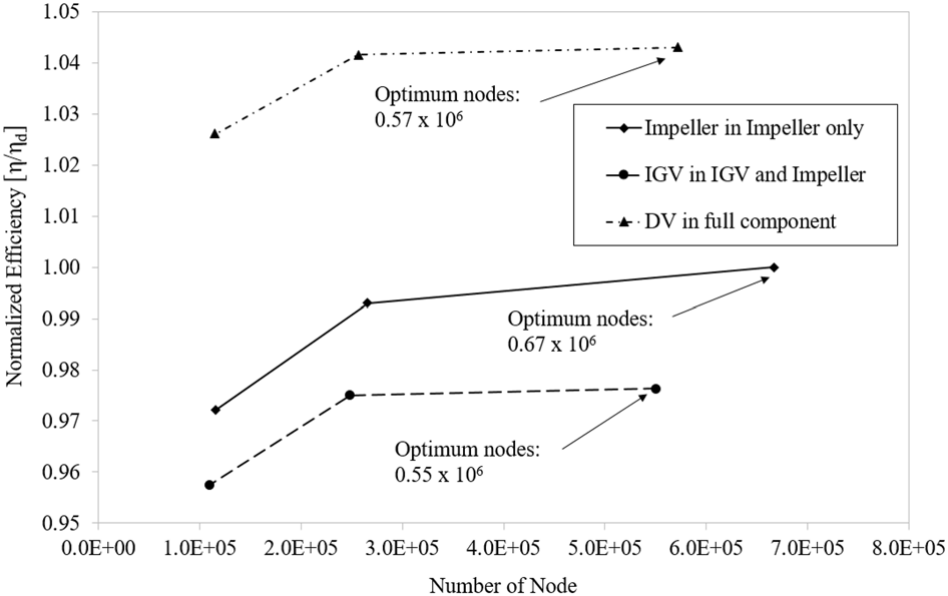
Comparison of efficiency for different grid sizes.

Figure 7 depicts a relationship between the efficiency of three different grid systems. The efficiency values are normalized by the value of efficiency in the fine grid system of the impeller part. In the case of impeller only, the difference in efficiency values between coarse and medium grid systems is approximately 2.096% and between medium and fine grid systems is approximately 0.71%. Similarly, these values for IGV passage are approximately 1.792% and 0.145%, respectively. These values for DV passage are approximately 1.505% and 0.13%, respectively. As the number of nodes increases, the difference between values in their efficiencies decreases. By a combination of GCI method results and the deviation values between the three different grids, the final number of nodes for the one IGV, one impeller, and one DV passages are 0.55 × 10^6^, 0.67 × 10^6^, and 0.57 × 10^6^, respectively.

## Results and discussion

### Numerical validation

**Figure 8:**
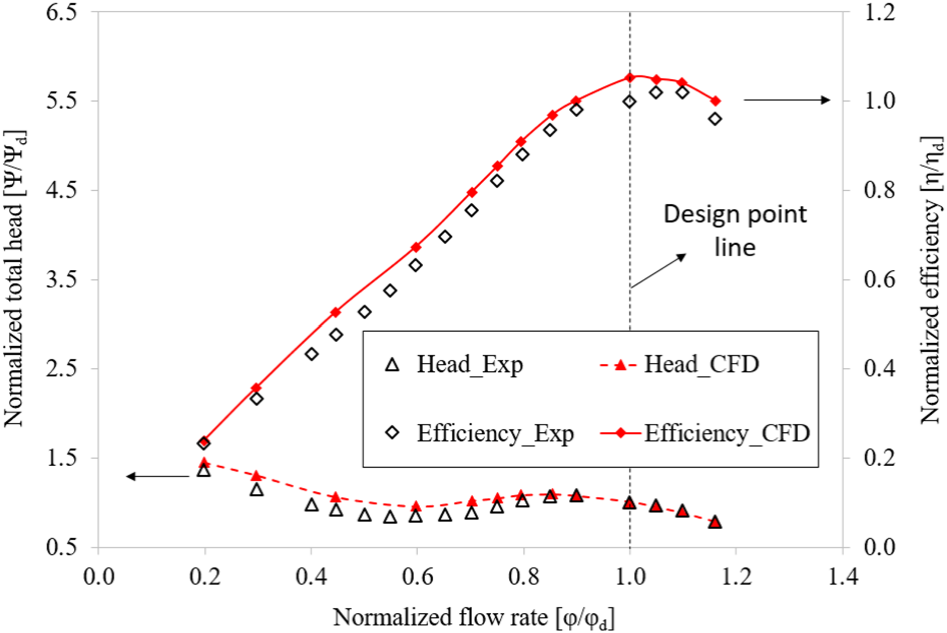
Hydraulic performance of the numerical and experimental results.

Figure 8 compares the hydraulic performance curves of the axial-flow pump between the experimental and numerical simulation results. The efficiency, total head, and flow rate values are non-dimensionalized based on their respective values at BEP from the experimental result. The obtained efficiency and total head curves of the experimental and numerical results are consistent and in good agreement with each other. At BEP, the total head of the numerical result is almost the same as the experimental result, whereas the efficiency is only 5.2% higher than the experimental result. These discrepancies are caused by measurement, equipment, mechanical loss, and CFD calculation errors. In addition, the direction of the inflow can affect the experimental and numerical results because the inflow for the numerical calculation in this case is straight. Under low flow rate conditions, the difference in total head between the two results is quite substantial because of the instability in flow that causes oscillations, resulting in a significant error. At the high flow rate region, the experiment yielded significant hydraulic losses due to long pipelines and numerous couplings in the test system without a booster pump. Therefore, in this study, the experimental results at the high flow rate region (larger than 1.2 ${\varphi }_{d}$) are unavailable. This validation confirmed the accuracy and dependability of the CFD results obtained in this study. As a result, the CFD method can be used to calculate new design models.

### Effect of inflow direction

**Figure 9:**
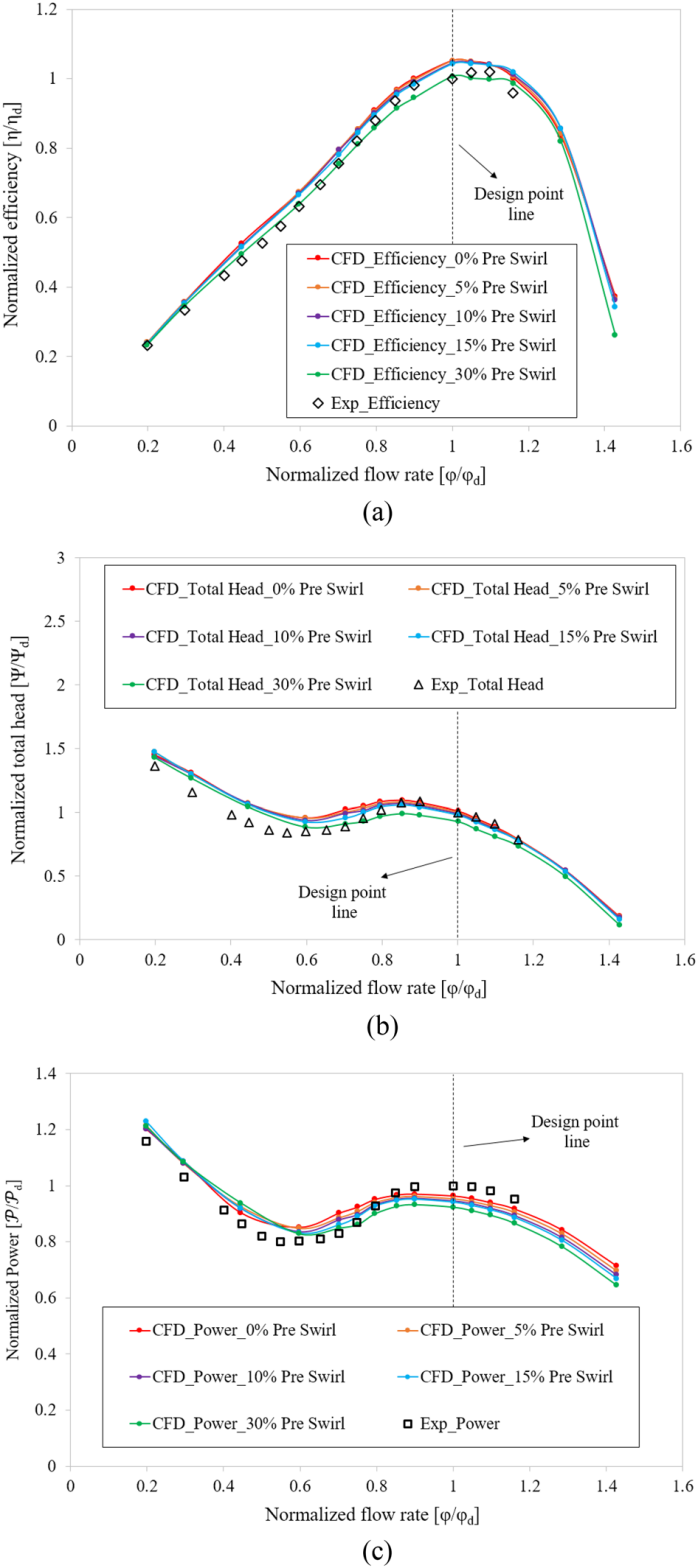
Comparison of hydraulic performance curves in experimental and numerical results with different pre-swirl intensities.

The real flow in front of the IGV is actualized through the pre-swirl flow at the inlet of the axial-flow pump. Figure 9 presents the external characteristic curves obtained by the CFD method for different pre-swirl intensities ranging from 0 to 30%. The total head, flow rate, efficiency, and shaft power values are non-dimensionalized by using their values at BEP of the experimental result. As can be observed from Fig. 9, the pre-swirl flow significantly affects the hydraulic performance of the axial-flow pump, and their relationship is inversely proportional. In other words, as pre-swirl intensity increases, head, efficiency, and power decrease, especially at high flow rates and in the saddle zone^15^. For pumps, a reduction in shaft power is beneficial; however, in this study, not only the shaft power decreased but also the total head and efficiency, especially at 30%. Therefore, the high pre-swirl intensity is ineffective for an axial-flow pump, where efficiency and head are prioritized. Consequently, the theoretical analysis and numerical simulation results are similar. The poorer performance before and after the design point is due to the instability in the internal flow caused by low and high flow rates. At the BEP, the efficiency decreased by 4.263% between the 0% and 30% pre-swirl intensity, while the total head and shaft power decreased by 8.267% and 4.183%, respectively. These significant drops at high pre-swirl intensities indicate a significant loss at the IGV passage.

Compared to the experimental results, the efficiency of the 30% pre-swirl intensity seems to be the most accurate, with a deviation at BEP of only 0.717%. However, at certain flow rates, such as at the saddle zone, the experimental result yields a higher efficiency than the numerical result. Several losses, such as mechanical losses, shock losses, and leakage losses, were included in the experimental results. Therefore, the efficiency of the experiment should be lower than the numerical result. In addition, comparing the total head and shaft power between the 30% pre-swirl intensity and the experiment shows that the compatibility is poor for the total head and shaft power, which are reduced by 7.114% and 7.644%, respectively, compared to the experimental result at BEP. Consequently, in this study, the inlet flow direction could not achieve 30% pre-swirl intensity.

**Figure 10:**
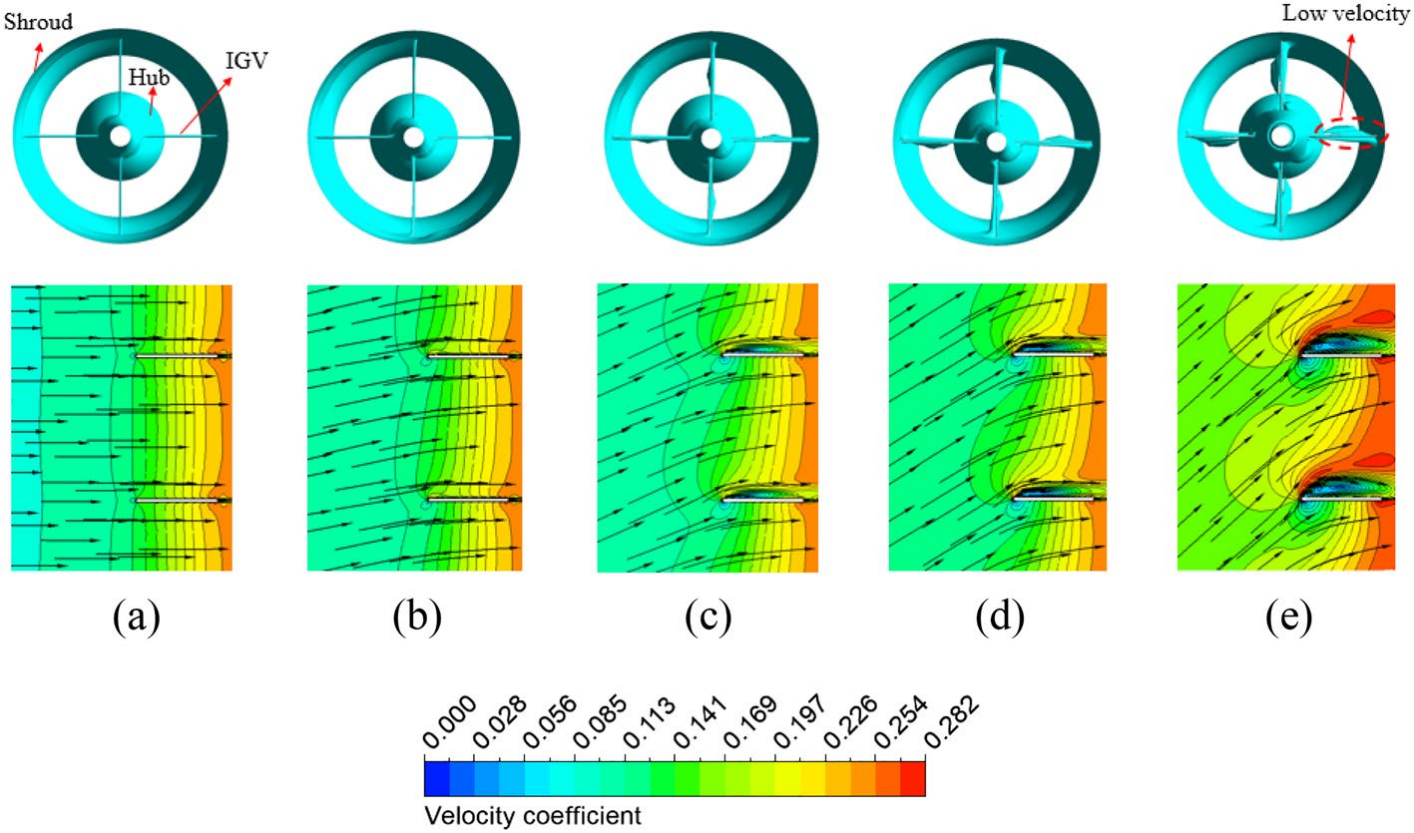
Low-velocity zone and velocity distribution at 50% span of the IGV passage with different pre-swirl intensities. (**a**) 0%, (**b**) 5%, (**c**) 10%, (**d**) 15%, and (**e**) 30%.

**Figure 11:**
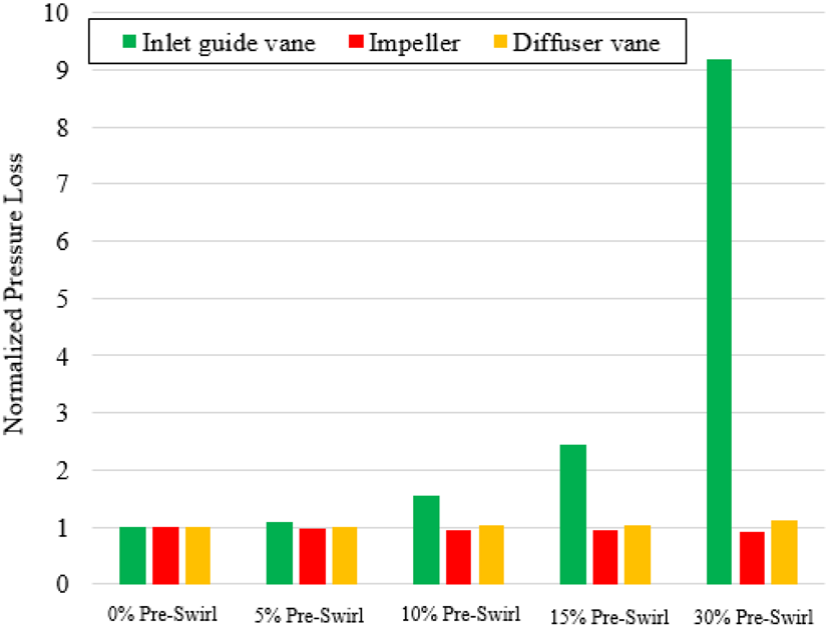
Pressure loss in the IGV, impeller and DV with different pre-swirl intensities.

In Fig. 10, the velocity coefficient distributions at 50% span and the low-velocity zone in the IGV passage show the low-velocity zones responsible for the hydraulic loss. The cyan color presents the low-velocity zone, with a velocity coefficient lower than 0.04. The velocity coefficient is defined by its ratio to the impeller tip velocity. The flow directions in front of the IGV obtained by changing pre-swirl intensity are shown with a black arrow in Fig. 10. As the pre-swirl intensity increases, the flow direction tends to become more inclined relative to the rotating shaft due to the increase in the swirl component of circumferential velocity. At high pre-swirl intensity, the low-velocity zone caused by flow separation and recirculation is formed at the SS of the IGV because of the mismatch between flow and blade angle at the LE of the IGV. The formation of the recirculation zone leads to a sharp increase in velocity, and the promotion of instability results in chaos. In addition, this resulted in an unequal velocity distribution at the front of the impeller. Due to the hydraulic loss at the IGV passage, the performance was dramatically reduced. To further explore the internal flow loss, the pressure loss proportion of each component of the axial-flow pump under different pre-swirl intensities is shown in Fig. 11. The pressure loss values are normalized by their values at 0% pre-swirl intensity. It can be observed that when the pre-swirl intensity increases, the pressure loss in the IGV passage increases, and the hydraulic performance gradually decreases. Particularly at 30% pre-swirl intensity, a significant drop in the performance of the axial-flow pump is caused by a significant pressure loss in the IGV passage. As a result, the fluid cannot smoothly flow through the IGV, complicating the flow entering the impeller. The total pressure loss is calculated as follows:

19 $$\Delta {H}_{s}= \frac{{P}_{in}- {P}_{out}}{\rho g},$$

20 $$\Delta {H}_{r}=\frac{{P}_{p}}{\rho gQ}-\frac{{P}_{out}- {P}_{in}}{\rho g},$$

where $\Delta {H}_{s}$, $\Delta {H}_{r}$, ${P}_{in}$, ${P}_{out}$, and ${P}_{p}$ are the total pressure loss in stationary domain, the total pressure loss in rotating domain, the total pressure at inlet (Pa) , the total pressure at outlet (Pa), and the input shaft power (W).

**Figure 12:**
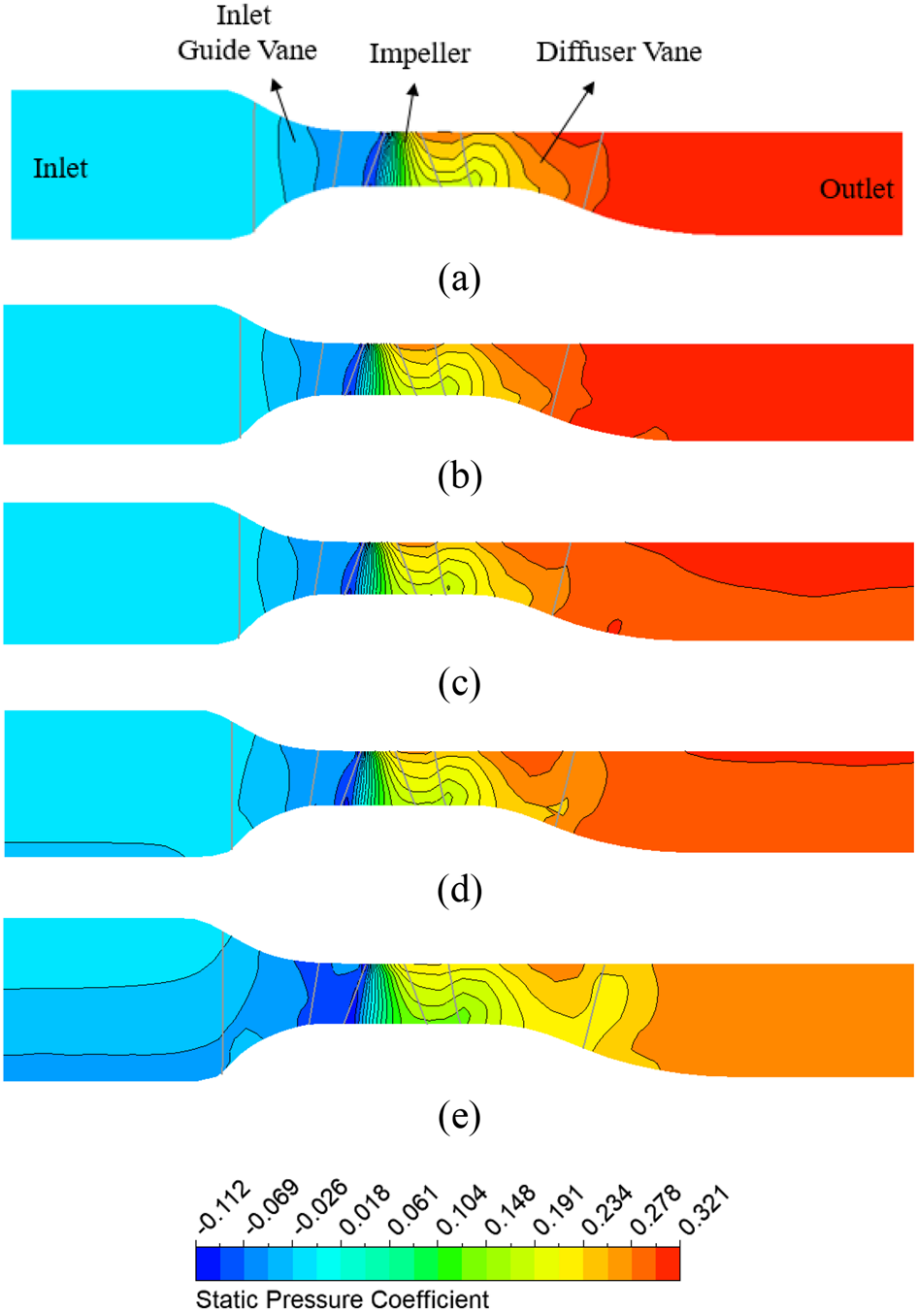
Static pressure distribution at meridional plane in different pre-swirl intensities. (**a**) 0%, (**b**) 5%, (**c**) 10%, (**d**) 15%, and (**e**) 30%.

Figure 12 compares the static pressure coefficient distributions on the meridional plane for different pre-swirl intensities. The static pressure coefficient $({C}_{P})$ is defined by Eq. (21), where *P* and *P*_in_ are static pressures. The pressure distributions at the front of the impeller are not uniform at high pre-swirl intensities because of the low-pressure zone. When combined with the chaos in the internal flow field, this low-pressure zone can produce bubbles that lead to the formation of the cavitation phenomenon at the LE of the impeller blade. The reduction in pressure at the IGV passage also decreases the static pressure of the impeller and DV passages. Due to the change in inflow direction, the flow characteristics inside the axial-flow pump are also varied. This altered the flow angle at the LE of the impeller and the trailing edge (TE) of the DV. As a result, at high pre-swirl intensity, a significant drop in static pressure is observed at the TE of the DV induced by the trailing edge vortex^5^. Therefore, the axial-flow pump with 30% pre-swirl intensity exhibits about 12.11% more pressure loss as compared to 0% pre-swirl intensity.

21 $${C}_{P}= \frac{P- {P}_{in}}{\frac{1}{2}\rho {V}^{2}},$$

where ${C}_{P}$, $P$, ${P}_{in}$ and $V$ are the pressure coefficient, the pressure, the averaged pressure at the inlet, and the velocity at the impeller’s tip, respectively.

**Figure 13:**
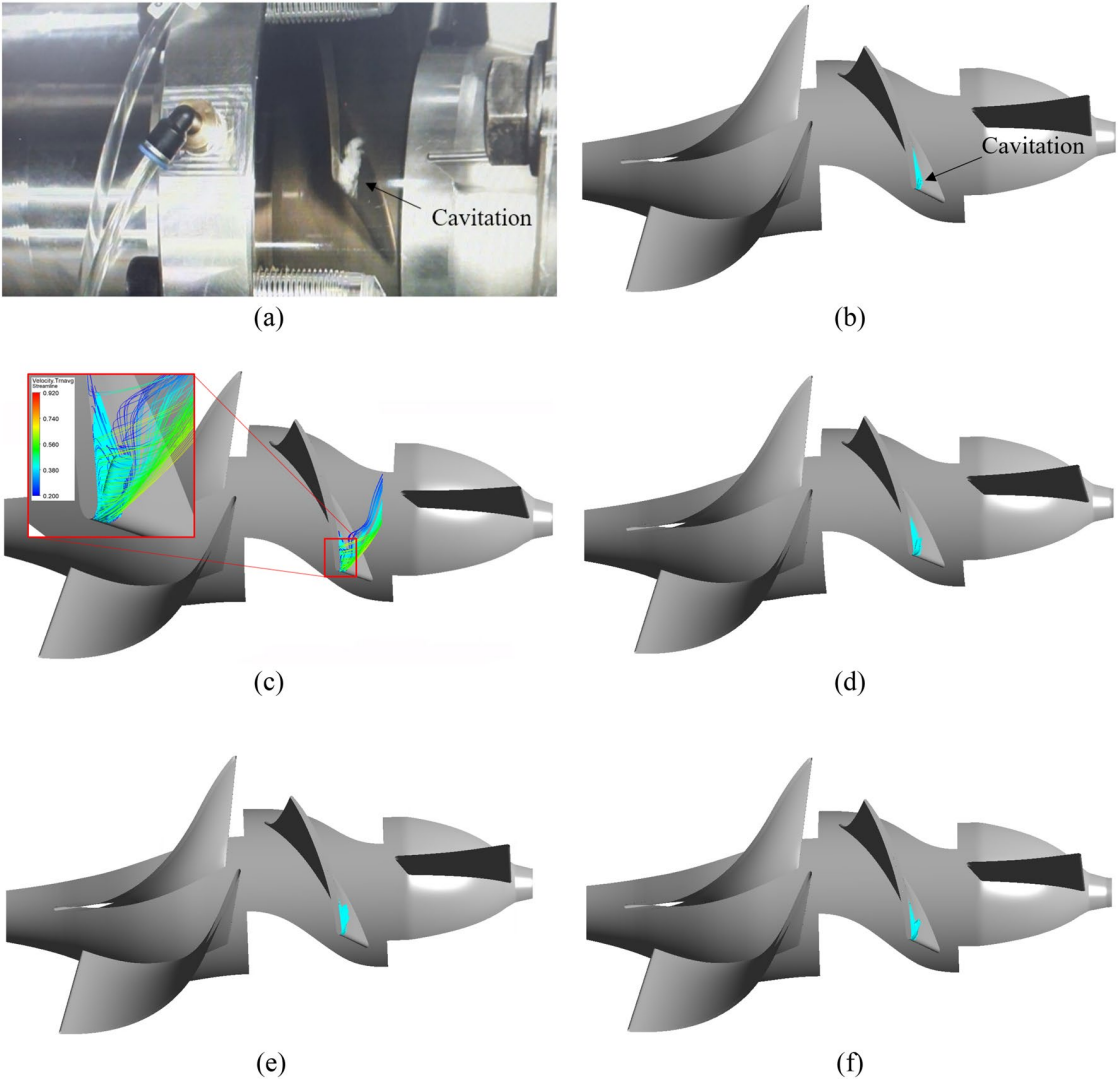
Internal flow comparison of experiment with different flow intensities in numerical result at 0.6 ${\varphi }_{d}$. (**a**) Experimental result, (**b**) 0%, (**c**) 5%, (**d**) 10%, (**e**) 15%, (**f**) 30%.

**Figure 14:**
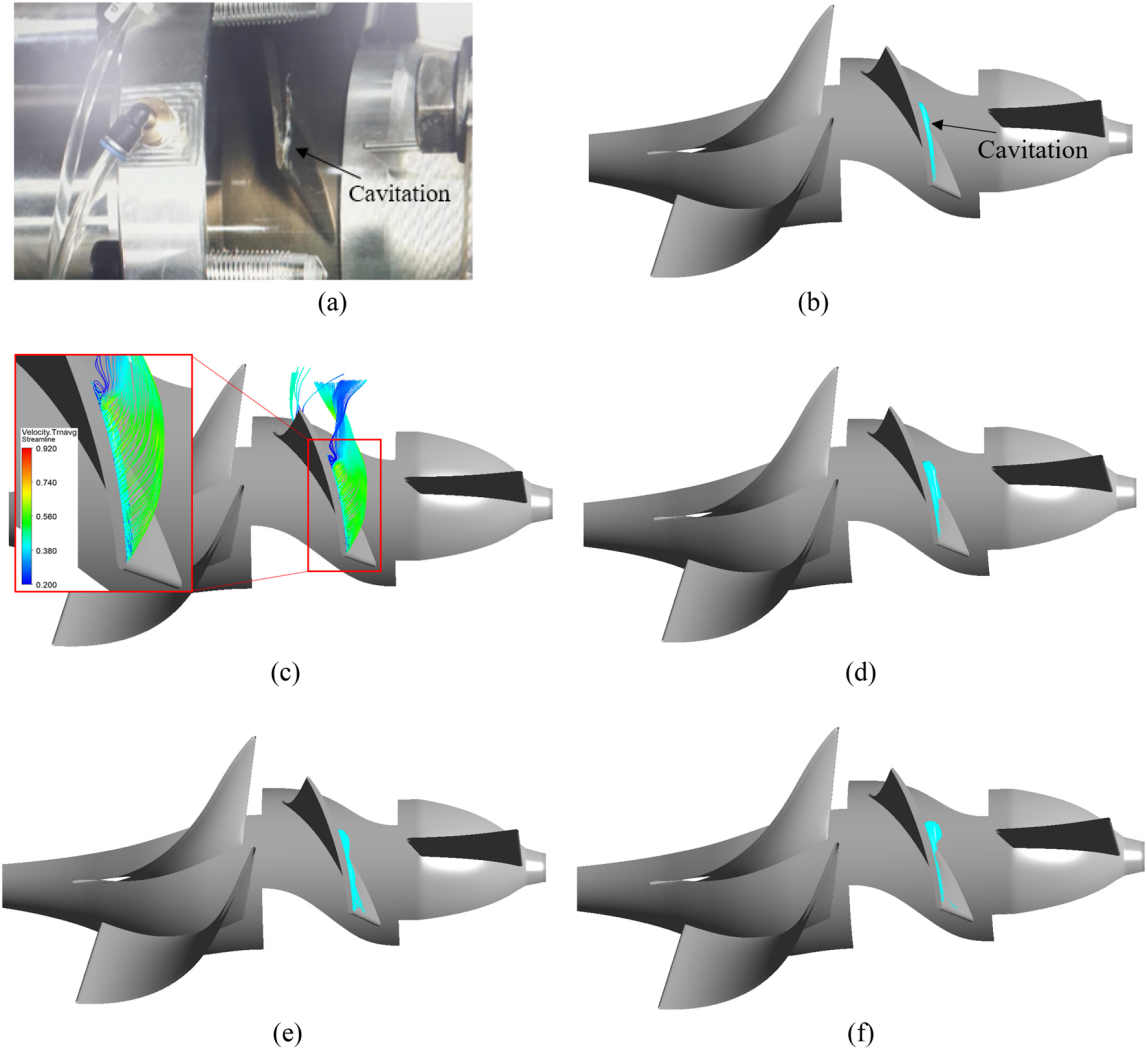
Internal flow comparison of experiment with different flow intensities in numerical result at 0.85 ${\varphi }_{d}$. (**a**) Experimental result, (**b**) 0%, (**c**) 5%, (**d**) 10%, (**e**) 15%, (**f**) 30%.

Figures 13 and 14 compare experimental and numerical results for the internal flow at the impeller of an axial-flow pump with different pre-swirl intensities at 0.6 ${\varphi }_{d}$ and 0.85 ${\varphi }_{d}$. In numerical results, internal flow is visualized by vapor volume fraction iso-surface. At 5% pre-swirl intensity for each flow rate condition, the averaged 3D velocity streamline coefficient transient is used to observe the formation and development of the cavitation. According to Kan et al.^15^, the flow rate of 0.85 ${\varphi }_{d}$ in this study is referred to as the critical stall point, whereas the flow rate of 0.6 ${\varphi }_{d}$ is known as the deep stall point. At these flow rates, bubbles appear and cavitation severely occurs, causing hydraulic losses that reduce the efficiency of the axial-flow pump. At the deep stall point, the experiment shows that many bubbles are formed at the LE of the impeller. These bubbles spread from around 80% span to the tip of the blade and its tail is separated from the blade surface because of the high incidence angle of the flow under conditions of low flow rate. At the critical stall point, cavitation is reduced, with a small number of bubbles forming at the tip of the blade and extending to around mid-blade. To ensure flow stability, the internal flow fields in the unsteady numerical simulation are obtained during the final revolution of the impeller. In the numerical results, where cavitation forms and grows is highlighted by a cyan color at the LE of the impeller. At the deep stall point, the cavitation size for 0% pre-swirl intensity is relatively tiny with only small TLV forming and a minor trail of bubbles adhering to the tip region of the blade surface. In addition, the cavitation shape for 15% and 30% pre-swirl intensities is inconsistent with experimental results because there is no separation of the cavitation from the blade surface in numerical results. At the deep stall point, it can be observed that 5% and 10% pre-swirl intensities produce cavitation results consistent with experimental results. The formation and evolution of cavitation at 0.6 ${\varphi }_{d}$ are significantly influenced by tip leakage flow and flow separation at the LE of the impeller. Combining these two types of flow creates a flow that separates from the SS of the impeller and rolls up to form the TLV^34^. Due to the high rolling velocity generated by the high velocity in both tip leakage flow and flow separation, a significant void forms in the TLV core. With the high velocity of the TLV, the pressure in this void is reduced below the saturation pressure, resulting in bubbles and cavitation.

At the critical stall point, the cavitation shapes obtained for 0% and 5% pre-swirl intensities are in good agreement with the experimental results, whereas 10% and 30% pre-swirl intensities present a large amount of cavitation appearing around mid-blade. The case of 15% pre-swirl intensity shows the formation of a trail of bubbles spreading toward the hub of the blade. The mechanism of formation and development of cavitation at 0.85 ${\varphi }_{d}$ is similar to that at 0.6 ${\varphi }_{d}$, but the intensity of cavitation diminishes because of a decrease in flow separation. It can be observed that a rolling flow appears at the tail of the cavitation with low velocity. Based on the comparison of experimental and numerical results at critical and deep stall points, as well as hydraulic performance curves, as shown in Fig. 9, it can be concluded that 5% is the suitable pre-swirl intensity for the inlet flow condition, and it will be used in the subsequent sections of this study. In addition, the IGV plays a vital role in directing the inlet flow. Without IGV, the high pre-swirl intensity at the inlet of the axial-flow pump will significantly decrease its hydraulic performance, especially at the off-design point.

**Figure 15:**
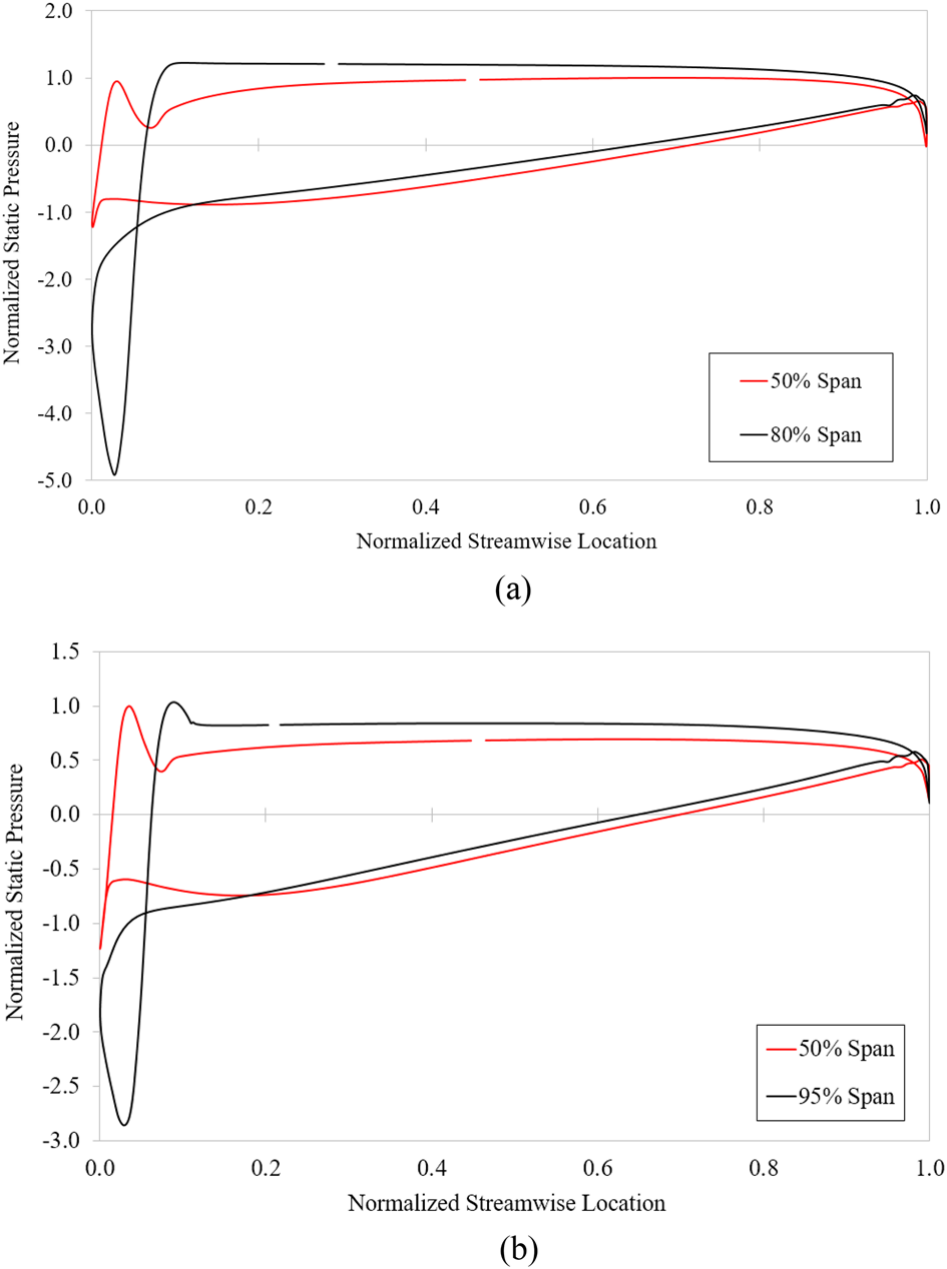
Blade loading distribution at different spans on the impeller at 5% pre-swirl intensity. (**a**) 0.6 ${\varphi }_{d}$ and (**b**) 0.85 ${\varphi }_{d}$.

Figure 15 presents the static pressure distribution at different spans on the impeller of the axial-flow pump at 5% pre-swirl intensity under 0.6 ${\varphi }_{d}$ and 0.85 ${\varphi }_{d}$ for insight into cavitation. The static pressure in each flow rate condition is normalized using the highest pressure value at 50% span. In both cases, the static pressure evenly distributes from the streamwise location range of approximately 0.1 to 0.95. However, the distribution is complicated at the LE and TE. Under deep stall condition, the pressure at the LE of the impeller at 80% span is dramatically lower than at 50% span. This sharp drop causes cavitation, which significantly reduces the hydraulic performance of the axial-flow pump. Similarly, under critical stall condition, the pressure drop at the LE of the impeller is observed to be greater at 95% span than at 50% span. However, the pressure reduction at the LE of the impeller under critical stall condition is relatively small compared to that under deep stall condition. Figure 15 also shows that cavitation under deep stall condition occurs right at 80% span while under critical stall condition is at 95% span. This explains why the hydraulic performance in deep stall condition is significantly lower than under critical stall condition. This sharp drop in pressure at the LE of the impeller also indicates that the incidence angle is too high, causing flow separation.

### Effect of clearance

**Figure 16:**
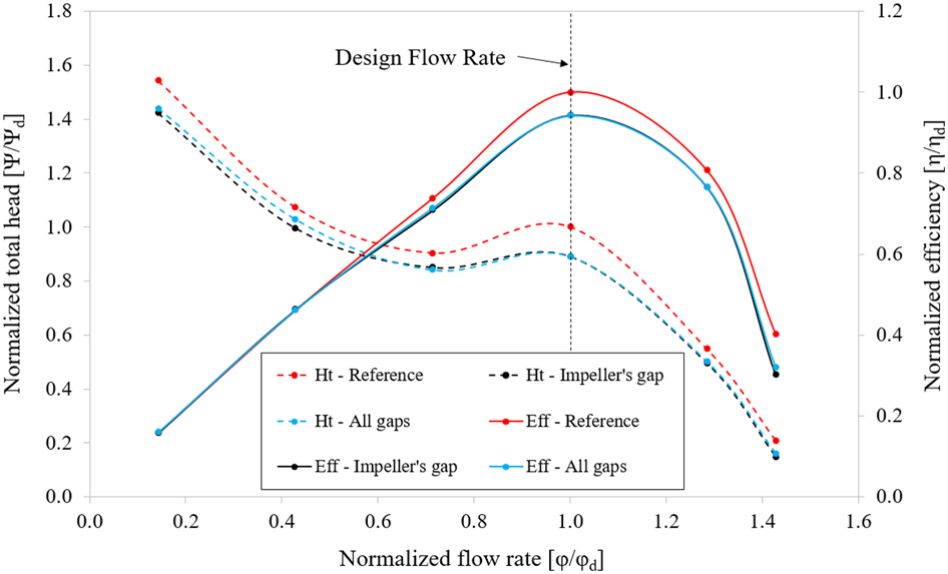
Hydraulic performance curves in different clearance types.

Creating clearance between the hub and shroud of the IGV is essential for adjusting the setting angle of the IGV. Figure 16 shows the efficiency and total head curves for three cases, including the tip clearance of the impeller case. The total head, flow rate, and efficiency values are correspondingly normalized by their values at the BEP of the reference case. The hydraulic performance is almost unchanged by the presence of the gap at the IGV compared with the case of only impeller gap. This is consistent with the hypothesis stated in the theoretical analysis, as the velocity in the IGV passage is relatively not complicated and the IGV blade is stationary. Notwithstanding, creating a large gap at the IGV can still cause a certain loss. In this study, the maximum gaps at the hub and shroud at the largest IGV setting angle (30°) were determined. Thus, IGV gaps greater than those measured will not be considered. The impeller tip clearance significantly decreases the hydraulic performance of the axial-flow pump right at the BEP. The efficiency and total head reduction are approximately 5.766% and 10.852%, relative to the reference case, respectively. The presence of the impeller clearance generates turbulence within the impeller passage, causing a drop in performance at the impeller and a general reduction in the overall efficiency of the axial-flow pump.

**Figure 17:**
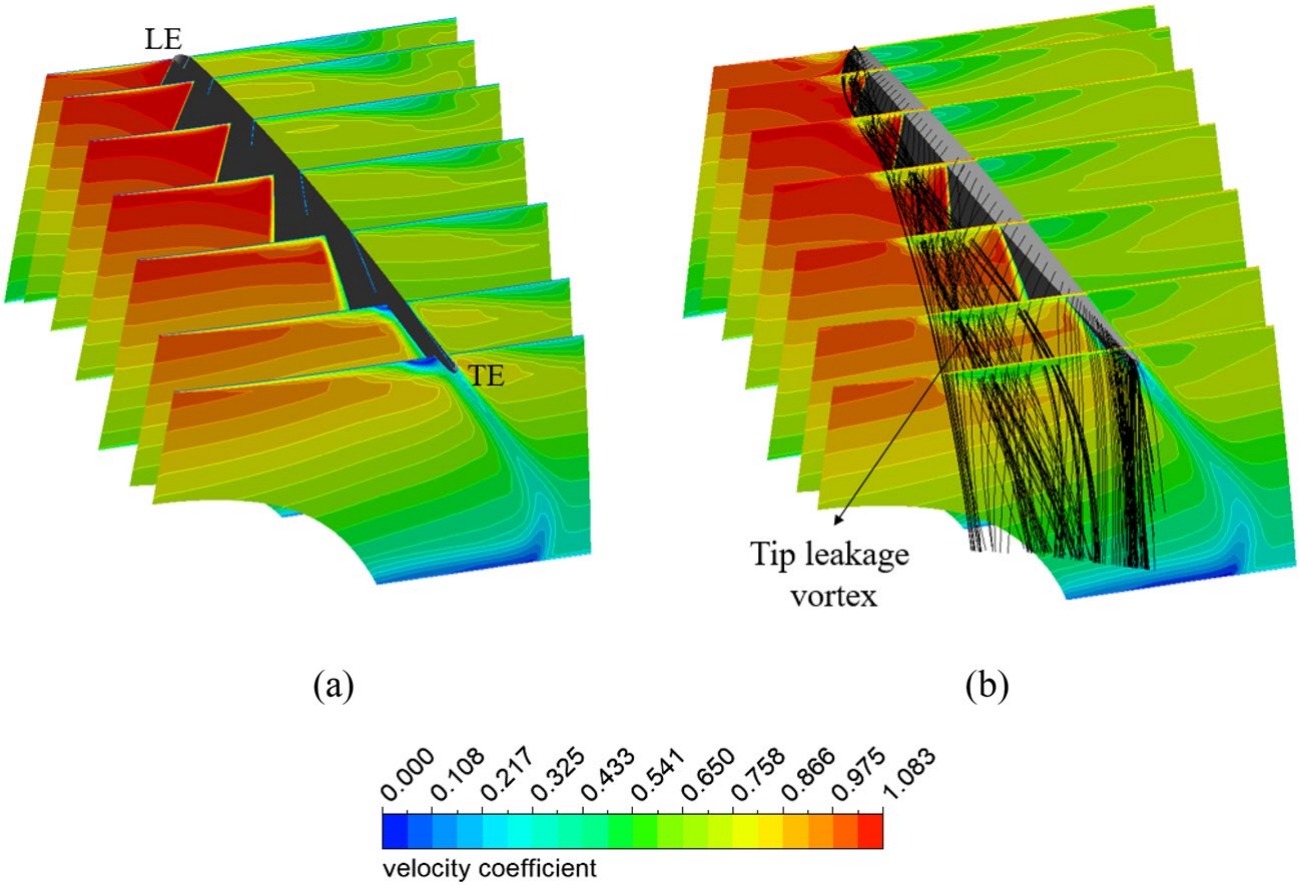
Velocity and streamline distribution in impeller passage. (**a**) Reference case (no gap) and (**b**) impeller’s clearance.

Figure 17 shows the velocity distribution in multiple planes and the streamline distribution at the clearance of the impeller to better visualize the generation and evolution of the vortex at the impeller tip clearance. Without clearance, the fluid flows smoothly from the LE to the TE of the impeller. With the appearance of the clearance, the fluid overflows from the PS to the SS at the tip of the impeller through the gap. This results in the generation of the vortex at the LE, where it is in direct contact with the flow. This vortex is accumulated and gradually grows from the LE to the TE and spreads in the passage of the impeller, causing a significant loss and affecting the internal flow characteristics. This vortex is often called the TLV^2^. In this study, since the tip of the impeller clearance is relatively small, the TLV that forms and develops tends to be closer to the shroud of the impeller. It can be observed that the TLV spreads to the adjacent impeller blade and causes chaos in the flow, resulting in bubbles being produced and possibly causing cavitation.

**Figure 18:**
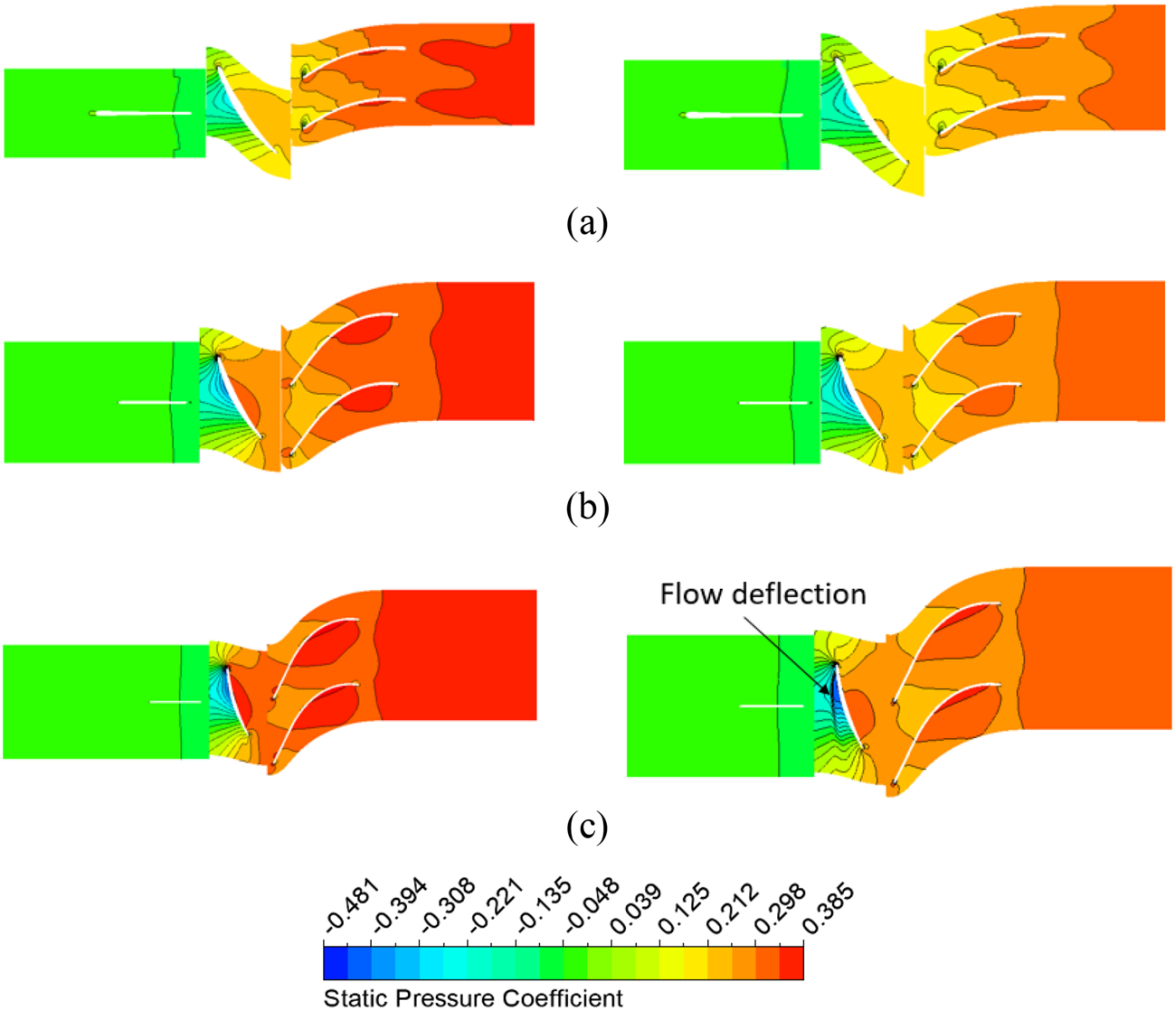
Static pressure distribution in reference case (left) and impeller’ gap case (right). (**a**) 5% span, (**b**) 50% span, and (**c**) 95% span.

The influence of the tip clearance of the impeller on the pressure is demonstrated by the static pressure coefficient distribution at 10%, 50%, and 90% span, as shown in Fig. 18. The static pressure coefficient is defined by Eq. (21), where both *P* and *P*_in_ are static pressures. The static pressure significantly decreased at the PS of the impeller. Meanwhile, the presence or absence of the IGV’s clearances does not affect the static pressure of the axial-flow pump. Hence, the reduction in the overall pressure of the pump primarily comes from the drop in pressure at the impeller passage. The pressure at PS of the impeller is significantly decreased because of the influence of TLV. At 95% span, the low-pressure area at the SS of the impeller is entirely covered by the TLV area. In addition, the evolution of the TLV causes a flow deflection at the impeller passage, affecting the near LE of the adjacent impeller blade.

### Effect of IGV’s setting angle

**Figure 19:**
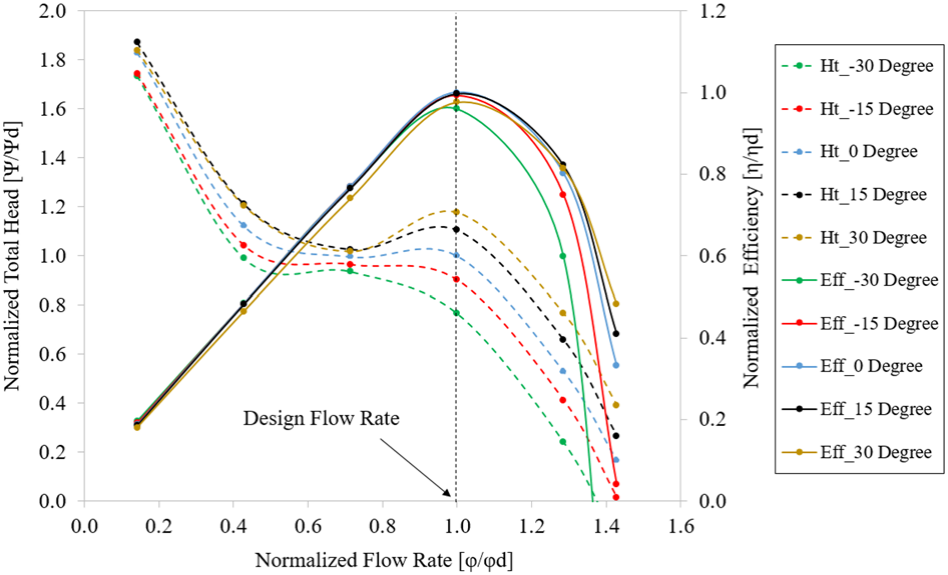
Hydraulic performance curves of axial-flow pump with different IGV’s setting angles.

This study proposes adjusting the setting angle of the IGV to reduce the impact of pre-swirl flow and improve hydraulic performance. Figure 19 depicts the hydraulic performance curves for different setting angles of the IGV. The total head, flow rate, and efficiency coefficients are normalized by their values at the BEP in the case of setting angle of the IGV at 0°. The hydraulic performance gradually improves by changing the setting angle of the IGV from negative to positive, particularly at high flow rates. However, the total head is no longer increased at low flow rate when the setting angle of the IGV is equal to 30°, even the efficiency is decreased at design point because of many vortices in the IGV passage and excessive power consumption. For this reason, 30° is considered the critical setting angle for IGV. From the IGV setting angle of − 30° to 30°, the total head at BEP gradually increases by approximately 10% in each case. When the IGV setting angle is increased, the saddle region is shown more clearly because of the loss at the LE of the IGV passage. Furthermore, the hydraulic performance is not much increased at low flow rates because of the losses caused by the flow separation at SS of the impeller due to the high incident angle at LE of the impeller at the conditions of low flow rate. There is a significant difference in efficiency under high flow rates due to the stabilization in the flow characteristics with a suitable flow angle at LE of the impeller. At the low flow rates, the efficiency is almost invariable as the chaos in the internal flow field enhances the load acting on the impeller blade and power consumption.

**Figure 20:**
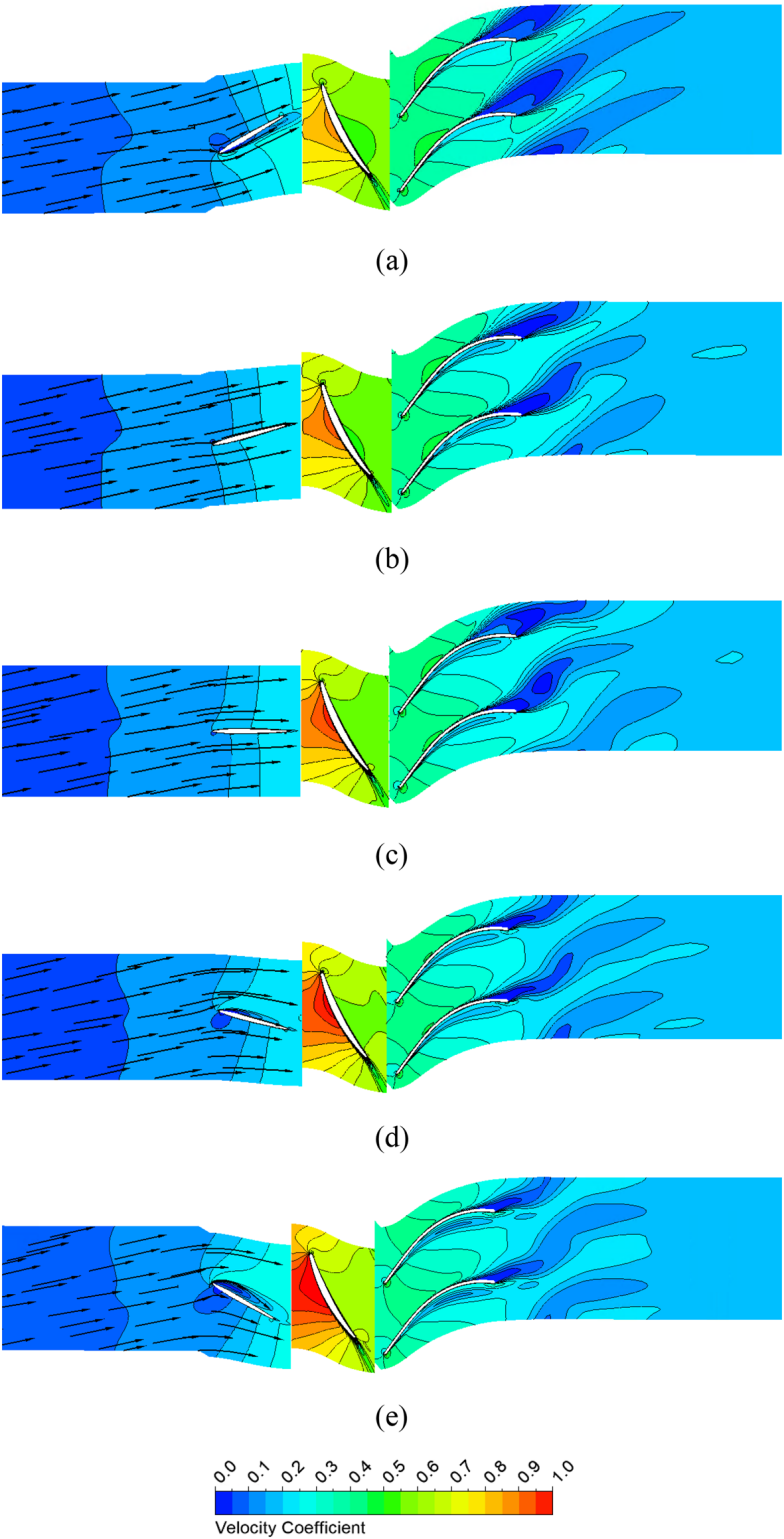
Velocity distribution at 50% span for various IGV’s setting angles. (**a**) − 30°, (**b**) − 15°, (**c**) reference case (0°), (**d**) 15°, and (**e**) 30°.

**Figure 21:**
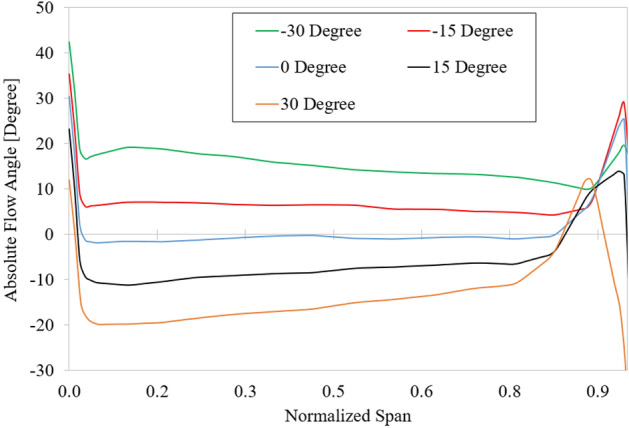
Absolute flow angle at leading edge of the impeller in different IGV’s setting angles.

The velocity vectors in the IGV passage and velocity coefficient distribution at a 50% span for various IGV setting angles are shown in Fig. 20. The velocity vector shows the fluid being redirected after flowing through the IGV blade with different setting angles. As a result, the hydraulic performance varies significantly among cases. Flow separation occurs at the LE of the IGV in the positive setting angle due to a mismatch between the flow and blade angle, causing a significant loss. Specifically, at a setting angle of 30°, despite the improvement in the performance of the impeller part, the entire hydraulic efficiency of the axial-flow pump is decreased due to a significant loss in the IGV passage. Nonetheless, the compatibility between the flow and blade angle at the LE of the impeller results in a significant increase in velocity for positive setting angles compared to negative setting angles, thereby enhancing the performance of the impeller passage. In cases of negative setting angle, despite good inflow conditions in the IGV passage, the hydraulic performance is significantly decreased relative to the reference case because of the high absolute flow angle at the LE of the impeller (as shown in Fig. 21). Again, the velocity distribution at the PS of the DV is unsatisfactory because of flow separation. Moreover, the trailing edge vortex^5^ is also observed at the TE of the DV, causing a decrease in the performance of the axial-flow pump. However, as the IGV’s setting angle increases, the internal flow characteristics change, resulting in a significant decrease in the area of the low-velocity region. This change is shown in Fig. 21 by the absolute flow angle at the LE of the impeller. As the IGV’s setting angle increases, the absolute flow angle gradually decreases, making the flow more compatible with the blade angle and increasing performance.

**Figure 22:**
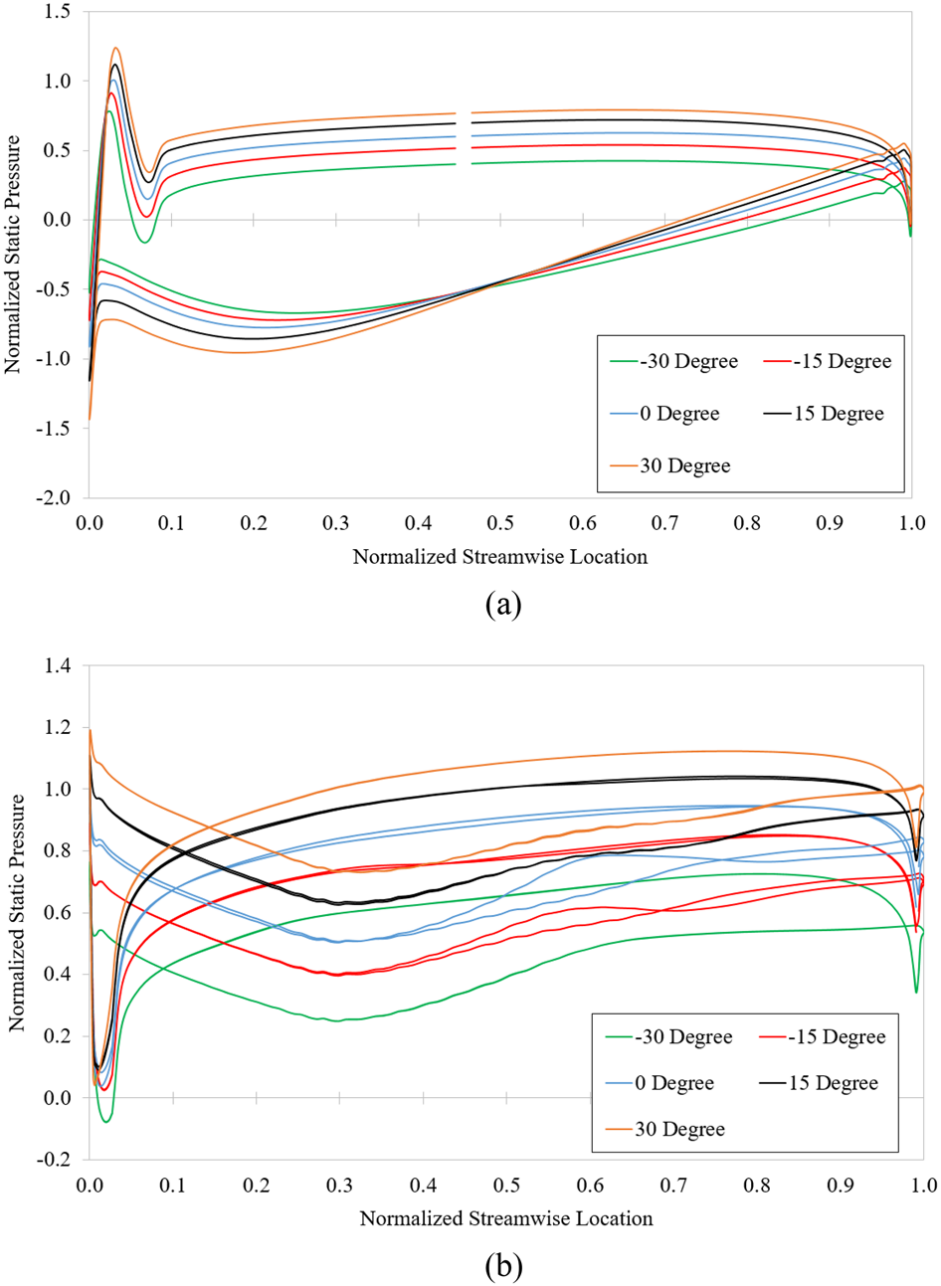
Blade loading at 50% span for various setting angles of IGV. (**a**) Impeller and (**b**) DV.

Figure 22a,b depict the blade loading distribution at 50% span of the impeller and DV, respectively. The pressure is non-dimensionalized using the maximum pressure value of the reference case. The pressure distribution gradually increases as the setting angle is increased. At the PS of the impeller, the pressure evenly rises at each streamwise location. There is a sharp enhancement in pressure at the LE of the impeller due to the appearance of the stagnation point. In addition, there is a significant drop in the pressure near the LE of the impeller because of the velocity acceleration on both sides of the impeller. Moreover, the large increment in velocity at the SS of the impeller results in a decrease in the pressure distribution from 0 to 40% streamwise location. The complication in pressure distribution at the TE of the impeller is caused by the flow separation^5^.

The increase in pressure in the DV mainly comes from the enhancement in pressure in the impeller passage. Not to mention, the change in internal flow characteristics also affects the pressure distribution of the DV. The pressure distribution trend of the DV is the same as that of the impeller, with the stagnation point at LE and the flow separation at TE. A sharp drop in pressure at the LE of the DV is caused by the influence of the low-velocity distribution of the adjacent blade and the acceleration in velocity at the LE of the DV, as shown in Fig. 20.

## Conclusion

This study shows the influence of inflow direction on the hydraulic performance of an axial-flow pump and the preventive measures that can be taken. The impact of the inflow direction is divided into five cases, with pre-swirl intensity ranging from 0 to 30%. The hydraulic performance curves and the internal flow characteristics are compared to the experimental results. To avoid the effect of the pre-swirl flow, it is proposed that the setting angle of the IGV be altered. The clearance at the hub and shroud is created to easily alter the setting angle of the IGV. The most extensive clearances in the IGV blade at the hub and shroud, as well as the clearance at the tip of the impeller, are investigated in detail. Meanwhile, five cases are being investigated to determine the impact of the setting angle for IGV. Based on the numerical results, the highlighted findings are summarized as follows:

1. The inflow direction significantly affects the hydraulic performance of an axial-flow pump. The inflow direction causes a difference between numerical and experimental results. As the pre-swirl intensity increases, the inflow becomes more inclined toward the rotating shaft, resulting in a mismatch between the flow and blade angle at the LE of the IGV. The performance gradually decreases as the pre-swirl intensity increases because of the loss at the IGV passage. The IGV is crucial in directing the inlet flow. Based on the hydraulic performance curves and internal flow characteristics, the 5% pre-swirl intensity is consistent with the experimental results. The mechanism of formation and evolution of the TLV is revealed with the combination of tip leakage flow and flow separation.
2. The influence of the clearance at the hub and shroud of the IGV on the hydraulic efficiency of the axial-flow pump is negligible. Due to the tip leakage flow, the tip clearance at the impeller significantly impacts the internal flow physics and performance of the axial-flow pump.
3. Adjusting the setting angle of the IGV alters the internal flow field and performance of the axial-flow pump. The hydraulic efficiency gradually increases as the setting angle transitions from negative to positive. At positive setting angles, there is a slight loss in the IGV passage due to the incompatibility between the flow and blade angle at the LE of the IGV. However, this loss is negligible when compared to the energy gained by the impeller. The increase in setting angle decreases the absolute flow angle at the LE of the impeller, enhancing the hydraulic performance of the impeller part. Nevertheless, the saddle zone becomes evident with positive setting angles. The critical point of the setting angle for the IGV is 30°.

In the future, experiments with particle image velocimetry will be used to examine the flow visualization and behavior of the inflow direction of the axial-flow pump in more depth. Experiments with different IGV setting angles will also be performed to verify the hydraulic performance of the axial-flow pump at various flow rates.

## Data Availability

The data that use and analyze in this study are available from the corresponding authors upon reasonable request.
